# Plant competition cues activate a singlet oxygen signaling pathway in *Arabidopsis thaliana*

**DOI:** 10.3389/fpls.2024.964476

**Published:** 2024-08-20

**Authors:** Nicole Berardi, Sasan Amirsadeghi, Clarence J. Swanton

**Affiliations:** ^1^ Ontario Ministry of Agriculture, Food and Rural Affairs, Guelph, ON, Canada; ^2^ Department of Plant Agriculture, University of Guelph, Guelph, ON, Canada

**Keywords:** acclimation response, *Arabidopsis thaliana*, low red to far-red ratio, plant competition, RNA sequencing, shade avoidance response, singlet oxygen signaling, jasmonate-dependent defenses

## Abstract

Oxidative stress responses of *Arabidopsis* to reflected low red to far-red signals (R:FR ≈ 0.3) generated by neighboring weeds or an artificial source of FR light were compared with a weed-free control (R:FR ≈1.6). In the low R:FR treatments, induction of the shade avoidance responses (SAR) coincided with increased leaf production of singlet oxygen (^1^O_2_). This ^1^O_2_ increase was not due to protochlorophyllide accumulation and did not cause cell death. Chemical treatments, however, with 5-aminolevulinic acid (the precursor of tetrapyrrole biosynthesis) and glutathione (a quinone A reductant) enhanced cell death and growth inhibition. RNA sequencing revealed that transcriptome responses to the reflected low R:FR light treatments minimally resembled previously known *Arabidopsis* ^1^O_2_ generating systems that rapidly generate ^1^O_2_ following a dark to light transfer. The upregulation of only a few early ^1^O_2_ responsive genes (6 out of 1931) in the reflected low R:FR treatments suggested specificity of the ^1^O_2_ signaling. Moreover, increased expression of two enzyme genes, the *SULFOTRANSFERASE ST2A* (*ST2a*) and the early ^1^O_2_-responsive *IAA-LEUCINE RESISTANCE (ILR)-LIKE6* (*ILL6*), which negatively regulate jasmonate level, suggested that repression of bioactive JAs may promote the shade avoidance (versus defense) and ^1^O_2_ acclimation (versus cell death) responses to neighboring weeds.

## Introduction

Singlet oxygen (^1^O_2_) is a potent oxidant that is generated in photosynthetic and non-photosynthetic tissues under multiple stresses (Dmitrieva et al., 2020). Several reports on the lifetime and diffusion distance of ^1^O_2_ in cellular environments indicate that ^1^O_2_ may diffuse through cell membranes (Dmitrieva et al., 2020; Ogilby, 2010; Hatz et al., 2007; Skovsen et al., 2005). Indeed, in *Chlamydomonas reinhardtii*, the photosystem II (PSII)-generated ^1^O_2_ under high light stress reached cytosol and induced expression of the glutathione reductase homologue GPXH (Fischer et al., 2007). These studies along with the detection of osmotic stress- and drought-induced ^1^O_2_ in *Arabidopsis* roots (Chen and Fluhr, 2018; Mor et al., 2014) indicate the possibility of ^1^O_2_ generation in cellular compartments other than chloroplast.

Under severe stress conditions, high levels of ^1^O_2_ can damage cellular components and impair plant function through photo-inhibition and uncontrollable cell death (Laloi and Havaux, 2015). At sub-lethal levels, however, ^1^O_2_ initiates signaling pathways that trigger disparate stress responses including acclimation to excess light and programmed cell death (op den Camp et al., 2003; Ledford et al., 2007; Laloi and Havaux, 2015; Waszczak et al., 2018; Ambastha et al., 2020).

Several plant systems allow for the study of ^1^O_2_ signaling. These include the conditional fluorescent (*flu*) mutant of *Arabidopsis* (Meskauskiene et al., 2001; op den Camp et al., 2003), the *tigrina* (*tig-d.12*) mutant of barley (Lee et al., 2003), and the *chlorina1* (*ch1*) mutant of *Arabidopsis* (Ramel et al., 2013a). The *flu* and *tig-d.12* mutants produce ^1^O_2_ in the light from dark-accumulated photosensitizer protochlorophyllide (Pchlide), whereas the *ch1* mutant of *Arabidopsis* and the *chlorina-f2* mutant of barley are deficient in chlorophyll b (Kim et al., 2009; Havaux et al., 2007; Havaux and Tardy, 1997; Leverenz et al., 1992). In the *ch1* mutant of *Arabidopsis*, PSII is confined to its reaction center due to the inability of PSII light-harvesting antennae to assemble without chlorophyll b. Without light-harvesting complex II (LHCII), PSII lacks photoprotective mechanisms like nonphotochemical quenching. Consequently, increasing photon flux density can lead to PSII overexcitation and the formation of ^1^O_2_ (Ramel et al., 2013a; Dall'Osto et al., 2010). While these mutant systems have provided valuable insight into the ^1^O_2_ signaling pathways, few plant systems allow the controlled induction of ^1^O_2_ and investigation of ^1^O_2_ signaling within wild type plants. Recently, ^1^O_2_ generation was detected in wild type *Arabidopsis* leaves following transfer of the FR light-treated seedling to white light (Page et al., 2017). This white light-mediated induction of ^1^O_2_ was due to Pchlide accumulation and led to suppression of major chlorophyll synthesis and photosynthetic genes presumably to prevent photo-oxidative damage during de-etiolation (Page et al., 2017).

Despite the detection of ^1^O_2_ in wild type *Arabidopsis* leaves following a short-time (two hours) FR light treatment and exposure to white light (Page et al., 2017), it is not clear whether ^1^O_2_ can be induced under low R:FR light environments. Such ^1^O_2_ induction may provide an opportunity to explore the involvement of ^1^O_2_ signaling as an intermediary between low R:FR light-mediated phytochrome inactivation and modulation of growth-defense trade-offs in response to competition cues. Under low R:FR light environments, phytochrome inactivation promotes growth-related hormonal pathways, while attenuating jasmonic acid (JA)-mediated defense responses (Ballaré, 2014; de Wit et al., 2016; Fernández-Milmanda and Ballaré, 2021). Attenuation of JA synthesis is also indispensable for triggering acclimation response to ^1^O_2_ and prevention of cell death, while ^1^O_2_-mediated photo-damage and cell death correspond with JA accumulation (Ramel et al., 2013a; Ramel et al., 2013b). Recently, a sulfotransferase (*ST2a*) has been shown to be up-regulated as a molecular link between low R:FR light environments and attenuation of JA-mediated defense responses through sulfation of bioactive JAs (Fernández-Milmanda et al., 2020). In addition, to a lesser extent, the low R:FR light upregulated the early ^1^O_2_-responsive amidohydrolase *ILL6* (op den Camp et al., 2003), which catalyzes the amido-hydrolysis of JA-isoleucine (Fernández-Milmanda et al., 2020). Given that Pchlide accumulation in the FR light-adapted plants leads to ^1^O_2_ production under white light (Page et al., 2017), a possibility arises that *ST2a* up-regulation in the low R:FR environments may be linked to ^1^O_2_-mediated signaling. In this work, wild-type *Arabidopsis* was used as a model to distinguish whether increased leaf production of ^1^O_2_ in response to competition cues is due to reflected far-red light from neighboring weeds and to gain insights into the ^1^O_2_ signaling under low R:FR light environments. Our findings under biological weedy and artificial sources of reflected low R:FR light show that elongation growth responses occur concurrently with ^1^O_2_ appearance suggesting its signaling role in response to competition cues. The ^1^O_2_ appearance is not a consequence of Pchlide accumulation and not sufficient to cause cell death but chemical treatments that increase Pchlide level or decrease chloroplast electron transport efficiency result in cell death and growth inhibition. The ^1^O_2_ signatures under the biological and artificial low R:FR light treatments differ dramatically from those in *Arabidopsis* mutants that rapidly generate ^1^O_2_ after dark to light transfer. Finally, the upregulation of *ST2a and ILL6* and the suppression of bioactive JAs may also operate in the acclimation to ^1^O_2_ under weed competition.

## Materials and methods

### Plant material and growth conditions

**Figure 1:**
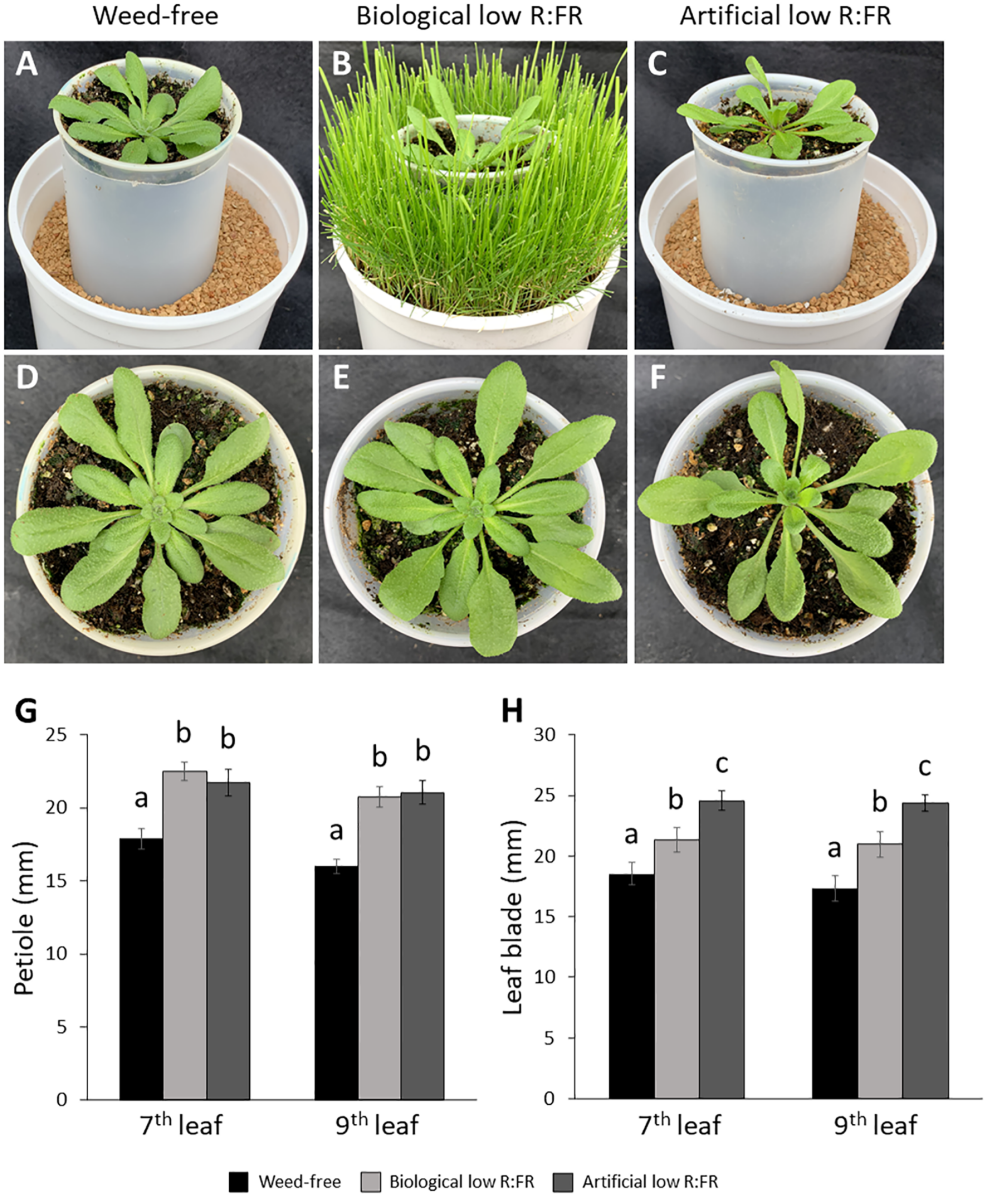
Weed-free control **(A)**, biological low R:FR **(B)**, and artificial low R:FR **(C)** treatments with reflected R:FR of ≈1.6, ≈0.3, and ≈0.3, respectively. *Arabidopsis* plants were exposed to the biological and artificial low R:FR light for 12 hours a day for seven days or kept under control (weed-free) condition prior to sampling. Plastic cups containing *Arabidopsis* seedling were placed in a plastic tube in the center of the pots to prevent direct root contact with neighbouring weeds. Note the hyponastic leaf growth in the biological and artificial low R:FR treatments. Normal leaf growth in the control **(D)**, and petiole elongation in the biological low R:FR **(E)**, and artificial low R:FR **(F)** treatments. Increases in petiole **(G)** and leaf blade **(H)** lengths of 7^th^ and 9^th^ leaf (4-week-old) in the low R:FR treatments. Black bars represent the control treatment while light grey and dark grey bars represent the biological low R:FR and artificial low R:FR light treatments, respectively. Note the similar elongation responses of *Arabidopsis* in separate growth chambers with the biological and artificial sources of reflected low R:FR light. Data represent means ± SEM for three independent experiments each consisting of five plants per treatment. Means were separated using Tukey’s HSD test (*P*<0.05). Letters indicate statistical significance of differences across treatments.

Wild-type *Arabidopsis thaliana* (ecotype Columbia) plants were raised in controlled environment growth chambers (Model CMP 3244 Conviron, Winnipeg, Canada) with a 12-hour photoperiod, an irradiance of 160 µmol m^-2^ s^-1^, a temperature of 21/18°C, and a relative humidity of 60%. The weed-free control (R:FR ≈ 1.6), biological low R:FR (R:FR ≈ 0.3), and artificial low R:FR (R:FR ≈ 0.3) light treatments were set up by placing plastic tubes (8 × 18 cm, 1 L) in the center of plastic pots (16 × 15 cm, 3.36 L) (Airlite Plastics Company, Omaha, USA). Drainage holes were drilled in the tubes and pots. For the control and the artificial low R:FR light treatments, the area between the plastic pot and plastic tube was filled with Turface MVP (Profile Products LLC, Buffalo Grove, USA). For the biological low R:FR treatment, the area between the plastic pot and plastic tube was filled with Sunshine Mix #4 (Sungro Horticulture, Agawam, MA) and seeded (≈ 200 g m^-2^) with a commercial mixture of grass seeds (The Scotts Compnay LLC, Marysville, USA). This mixture of grass seeds consisted of perennial ryegrass (*Lolium perenne* L.), creeping red fescue (*Festuca rubra* L.), Kentucky bluegrass (*Poa pratensis* L.), and chewing fescue (*Festuca rubra* L.) (The Scotts Compnay LLC, Marysville, USA). The grass was watered twice a week and was fertilized every two weeks with a nutrient solution as described previously (Tollenaar, 1989). A dense grass was established within two months and generated a stable biological source of reflected FR light without direct contact between the grass and experimental plants. A simulated FR light environment (artificial low R:FR; R:FR ≈ 0.3) was generated using 13 W, 162 mA far-red LEDs (Phillips Canada). Seeds of *Arabidopsis* were planted in a mix of PGx (Premier Horticulture LTD, Quebec, Canada) and perlite (Perlite Canada Inc., Quebec, Canada) at a ratio of 3:1 in 355 mL (8×10 cm) plastic cups (Dart Container Corp., Mason, USA). The plastic cups were then placed inside the plastic tubes in each pot in the respective light environments (**Figure 1**). The central plastic tube acted as a barrier preventing water exchange and nutrient flow between the *Arabidopsis* plants in the cups and the grass. Further, the empty space between the bottom of the cup and bottom of the central tube prevented secretion of grass metabolites to *Arabidopsis* roots. Light interference in the growth chambers was eliminated using a white opaque plastic divider between the weed-free control and biological low R:FR while allowing a free upward air flow (1.55 m^3^ min^-1^) across treatments. *Arabidopsis* plants were grown under weed-free control conditions for 21 days and fertilized weekly with a modified Hoagland’s solution (10 mM KNO_3_, 10 mM Ca(NO_3_)_2_.4H_2_O, and 2.5 mM KH_2_PO_4_). *Arabidopsis* plants were then exposed to either the biological or artificial low R:FR light treatment for 12 hours a day for seven days or kept under control conditions before plants were sampled. The light spectral composition of the weed-free control, biological low R:FR, and artificial low R:FR light treatments were determined at plant height. The incoming and reflected light quantity and quality were measured at nine locations across each treatment using a LI-COR-180 spectrometer (Li-COR Biosciences; Lincoln, NE, USA). For incoming light measurements, the spectrometer was held level at plant height facing the lights. For reflected light measurements, the spectrometer was held level facing the plants. A summary and detailed information about the light spectral composition in the weed-free control, biological low R:FR and artificial low R:FR light treatments are presented in **Supplementary Table 1**.

### Singlet oxygen imaging

Singlet oxygen formation was detected in 4-week-old *Arabidopsis thaliana* leaves using Singlet Oxygen Sensor Green (SOSG) (ThermoFisher, Waltham, USA). A 500 µM stock solution of SOSG was prepared by dissolving 100 µg of SOSG in 330 µL of methanol and diluted to a working concentration of 10 µM SOSG using 50 mM potassium phosphate buffer (pH 7.5) and 0.01% Tween-20 as a non-ionic surfactant. Plants were manually infiltrated with the 10 µM SOSG solution using a 60 ml needleless syringe and placed back in the treatment for two hours. The SOSG fluorescence (excitation ~450-490 nm; emission ~500-550 nm) was detected using an Axio Zoom V16 fluorescence stereo microscope with a Pan NeoFluor Z 1x/0.25 FWD 56 mm lens and a 38 HE filter set (Zeiss Canada, Toronto, Canada). All leaf images were obtained using the same magnification and an exposure of 500 milliseconds. The SOSG fluorescence signal was quantified using the image analysis application Fiji (Schindelin et al., 2012). Briefly, the fluorescence signal in each leaf image was quantified in five equal (200 × 200) regions of interest (ROIs) and averaged to obtain the mean fluorescence signal. For each treatment, 25 independent images were analyzed.

### Pigment analysis

To quantify photosynthetic pigments, leaf discs (nine mm) were taken from the mid-section of fully expanded *Arabidopsis* leaves (4-week-old) and placed in 1.5 mL of 80% acetone. The leaf discs were incubated in the dark at -20°C for 24 hours until total removal of chlorophyll. Absorbance was measured at 470, 626, 645, 646, 647, 663, and 664 nm. The concentration of Pchlide, chlorophyllide a (Chlide a), chlorophyll a (Chl a), chlorophyll b (Chl b), and total carotenoids were calculated using the previously described equations (Brouers and Michel-Wolwertz, 1983; Lichtenthaler and Wellburn, 1983). A more detailed quantification of individual carotenoids including lutein, β-carotene, violaxanthin, and neoxanthin was performed by high performance liquid chromatography (HPLC). Approximately, 20 mg of fresh ground tissue was used for HPLC analysis. To extract prenyl lipids, 250 µL of acetone:ethyl acetate (3:2; v/v) was added to each sample followed by the addition of 100 µL of ethyl acetate and 200 µL of dH_2_O. Each sample was then vortexed for five seconds and centrifuged (10 seconds burst) to achieve phase separation. The upper ethyl acetate layer was transferred into a vial and 50 µL of the ethyl acetate layer was separated on a five µm Spherisorb ODS-2 reverse-phase column (250 × 4.6 mm, Supelco) thermostated at 23°C. A linear gradient from 100% acetonitrile:water:trimethylamine (9:1:0.01) to 100% ethyl acetate was used to elute the samples at a flow rate of 1 mL min^-1^ over 45 minutes. Lutein, β-carotene, violaxanthin, and neoxanthin were detected at *A*_440_ with a detection limit of 0.05 nmol and were quantified based on external calibration standards of high purity. For these HPLC standards, lutein was obtained from Cayman Chemicals (Ann Arbor, MI, USA). β-carotene, violaxanthin, and neoxanthin were obtained from Sigma Aldrich (Oakville, ON, Canada).

### Chemical treatments

All chemicals were made up in 10 mM MES buffer (pH 6.5) and 0.01% tween-20 as a surfactant. The final concentrations of chemicals were 10 mM ALA (5-aminolevulinic acid; the precursor of tetrapyrrole biosynthesis), 50 mM, 100 mM, 150 mM, and 200 mM GSH (reduced glutathione; a quinone A reductant), and 50 mM, 100 mM, 150 mM, and 200 mM H_2_O_2_ (hydrogen peroxide; an oxidizing agent). For the ALA treatment, the upper and lower leaves of 4-week-old *Arabidopsis* plants were sprayed with a two mL solution of ALA. In total, four experiments were performed and ALA was sprayed on three plants per treatment per experiment. For the GSH and H_2_O_2_ treatments, the rosette leaves of 3-week-old *Arabidopsis* plants were sprayed with a one mL solution of GSH or H_2_O_2_. After spraying, plants were allowed to dry for one hour before transfer back to the treatments. The extent of cell death by ALA was monitored and imaged every 24 hours for a total of 72 hours. The extent of cell death by GHS and H_2_O_2_ was monitored every 24 hours and imaged 96 hours after spraying. In total, three independent experiments with a similar time course were performed and each chemical was sprayed on five plants per treatment per experiment. In separate experiments, the extent of cell death by GSH was quantified in 18 plants per concentration per treatment using the image analysis application Fiji (Schindelin et al., 2012). Dead leaf area was calculated by subtracting the green leaf area from total leaf area and expressed as percentage of leaf cell death.

### RNA-sequencing

RNA extraction from 4-week-old *Arabidopsis* rosette leaves and subsequent DNase treatment of RNA samples were performed using a RNeasy PowerPlant kit (Qiagen) and an RNase-Free DNase kit (Qiagen) according to the manufacturer’s instructions. In total, nine samples consisting of three treatments (weed-free, biological low R:FR, and artificial low R:FR light) with three replicates, each consisting of a pool of leaves from three individual plants, were analyzed. The RNA quality and quantity were determined using an Agilent 2100 Bioanalyzer (Agilent Technologies). The RNA libraries were constructed using the Illumina TrueSeq RNA kit in three replicates according to the manufacturer’s protocol. The RNA libraries were sequenced on an Illumina sequencer (NovaSeq 6000) at the Genome Quebec Innovation Center (McGill University, Canada) to obtain ≈25 M reads per replicate. Differentially expressed gene (DEG) analysis was performed by Harvest Genomics Inc. (Guelph, Canada), where the quality control of raw.fastq files was performed prior to sequence alignment to the reference genome (https://www.ncbi.nlm.nih.gov/assembly/GCF_000001735.4/) using Bowtie 2 v2.4.3. Count data were retrieved using HTSeq (version 0.13.5) and DEGs were determined using DESeq2 (version 1.30.1). The Benjamini-Hochberg false discovery rate (FDR) correction was used within DESeq2 (version 1.30.1) to obtain adjusted *p*-values (*p*adj). Tables of log_2_ fold change (lfc) were generated as described previously (Zhu et al., 2019). The RNA-seq raw reads and expression analysis are available in the NCBI gene expression omnibus (GEO) data repository under accession GSE213185 (https://www.ncbi.nlm.nih.gov/gds/?term=GSE213185).

### Real-time quantitative reverse transcription PCR assays

Total RNA was extracted from 4-week-old *Arabidopsis* leaves using the TRI Reagent (Sigma Aldrich Canada). In total, 16 samples from two independent experiments were analyzed. In each experiment, eight samples were taken from two treatments (weed-free and biological low R:FR light) each consisting of rosette leaves from four individual plants. The DNAse treatment and sample cleanup were performed using the RNAase-Free DNase Set (Qiagen Inc. Canada) and the RNeasy MinElute Cleanup kit (Qiagen Inc. Canada), respectively. Prior to RT-qPCR assays, RNA quality was determined using an Agilent 4150 TapeStation System (Agilent, CA, USA) according to the manufacturer’s instructions. The RNA samples were reverse transcribed using the High Capacity cDNA Reverse Transcription kit following the supplied protocol (Applied Biosystems, Canada). Real-time PCR assays were performed using a QuantaStudio Real Time PCR system (Thermo Fisher Scientific Inc., Canada). Each PCR reaction (20 μl) consisted of 10 μl of 2× SsoAdvanced Universal Inhibitor-Tolerant SYBR supermix (Bio-Rad, Cat No: 172-5017), 0.8 µl of PCR forward and reverse primer mix at 5 µM (final concentration of primer at 200 nM), 4.2 μl of water and 5 µl of 8× diluted cDNA. The thermal cycler conditions were 3 minutes at 98°C polymerase activation step, followed by 40 cycles of a two-step qPCR (10 seconds of 98°C denaturation, 30 seconds of 60°C combined annealing/extension). The amplified PCR products were compared with the *PROFILIN1* (*PRF1*) as a housekeeping gene and relative changes in gene expression were quantified using the 2^-ΔΔct^ equation (Livak and Schmittgen, 2001). Primers were designed using PrimerQuest Tool (Integrated DNA Technologies, Coralville, USA). The primer sequences used for qPCR were: *SULFOTRANSFERASE 2A* (At5G07010) *ST2a* fwd 5’-ACCTCAAGCATGAAGAGCATTC-3’ and *ST2a* rev 5’-CCCTTCATCTTCTTCGGCTTTC-3’; *SULFOTRANSFERASE 2B* (At5G07000) *ST2b* fwd 5’-AAGCGAAGGCCAAGAAGAA-3’ and *ST2b* rev 5’-GTAACGATTTCTCCGTCCTCTC-3’; *IAA-LEUCINE RESISTANCE (ILR)-LIKE6* (At1g44350) *ILL6* fwd 5’-TCTTGGTGCTGCCCATATTC-3’ and *ILL6* rev 5’-AAGCTCCGTCTTCGATCATATTC-3’; *U-BOX E3 UBIQUITIN LIGASE* (At3g19380) *PUB25* fwd 5’-CGACTTCACACTCATCCCTAAC-3’ and *PUB25* rev 5’-CAGCTGGTTGTTTAGGAGTAGG-3’; *PRF1* (At2G19760) fwd 5’-GGTGAACAAGGAGCTGTGAT-3’ and *PRF1* rev 5’-GGTTCATCGTAGAAGCCAAAGA-3’.

### Statistical analysis

Statistical analysis was performed using SAS version 9.4 and PROC GLIMMIX. The experiments were arranged as a randomized complete block design. The light treatment was considered the fixed effect, while replication was considered the random effect. Least-square means were generated, and means were separated with a Tukey’s honest significant difference (HSD) test. Unless stated otherwise, each replicate consisted of five *Arabidopsis* samples per treatment and three replications per experiment. A type I error of 0.05 was used for all tests of significance. A power analysis was performed to ensure adequate power was achieved.

## Results

### The shade avoidance response is accompanied by an increase of ^1^O_2_ level in leaves

**Figure 2:**
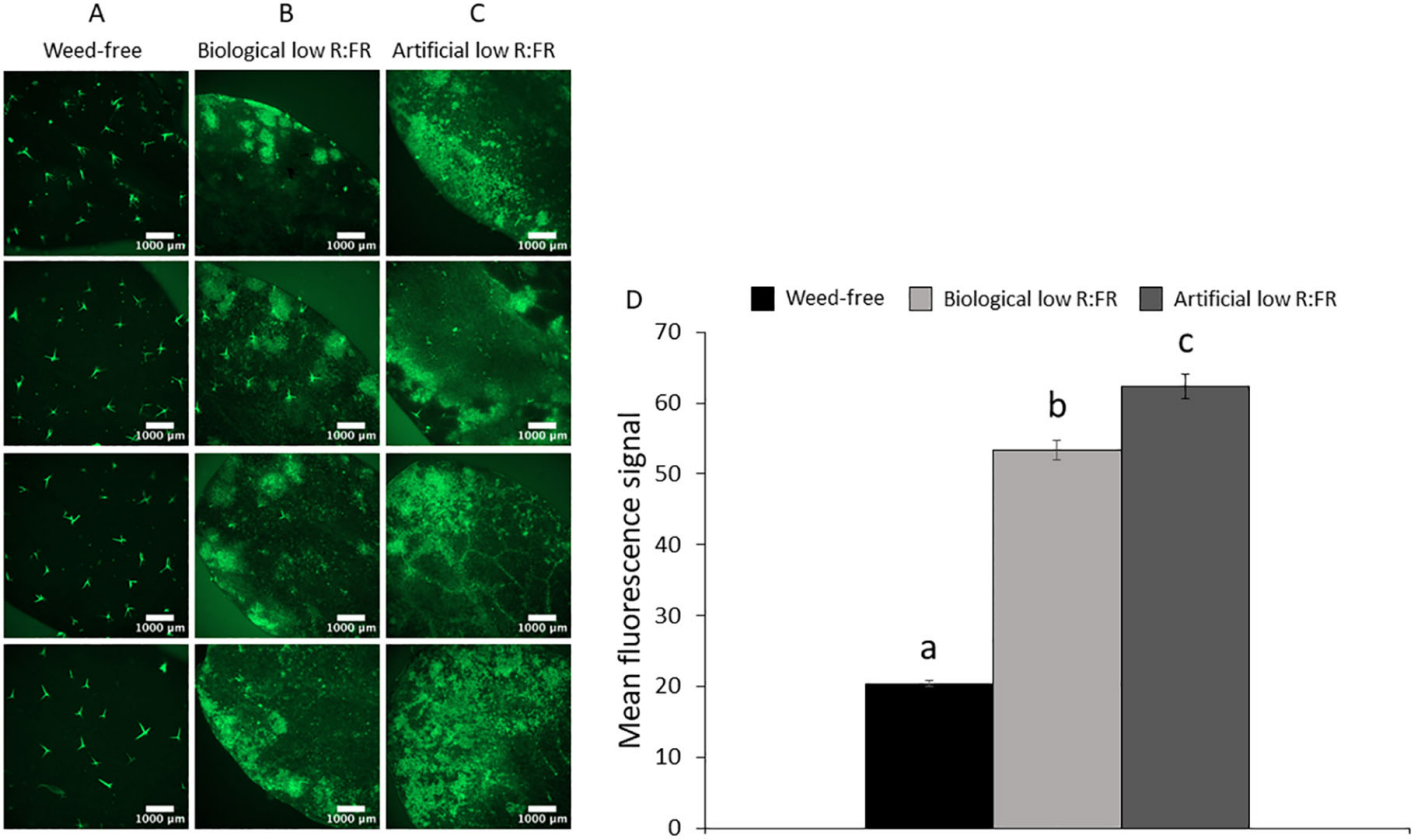
Basal level of ^1^O_2_ in the weed-free control **(A)** and increases in ^1^O_2_ levels in the biological low R:FR **(B)** and artificial low R:FR **(C)** treatments. Each panel shows four representative images of independent leaf samples. Four-week-old *Arabidopsis* plants were infiltrated with 10 µM SOSG and the SOSG fluorescence was imaged two hours after infiltration at an exposure time of 500 milliseconds. Scale bar represents 1000 μm. Significant increases in mean fluorescence signal in the biological and artificial low R:FR treatments **(D)**. The SOSG fluorescence was quantified using the image analysis application Fiji (Schindelin et al., 2012). Data represent means ± SEM for 25 independent leaf samples per treatment (**Supplementary Table 2**). Means were separated using Tukey’s HSD test (*P*<0.05). Letters indicate statistical significance of differences across treatments.

We exposed 3-week-old *Arabidopsis* plants to the biological and artificial low R:FR light for 12 hours a day for seven days or kept them in the weed-free control condition. This exposure to reflected low R:FR light was under resource-independent competition, where incoming light, water and nutrients were not limiting factors. Further, we prevented direct root contact between the *Arabidopsis* plants and the surrogate weeds by a plastic tube in the center of the pots (**Figures 1A–C**; **Supplementary Table 1**). Elongation growth and leaf hyponasty were typical shade avoidance responses (**Figures 1A–F**). The biological and artificial low R:FR treatments caused similar elongation responses in the petiole lengths (23% and 26%) and leaf blade lengths (14% and 19%) of 4-week-old *Arabidopsis* plants (**Figures 1G–H**). These shade avoidance responses were accompanied by increases in leaf production of ^1^O_2_ in the biological and artificial low R:FR treatments (**Figures 2A–C**). Quantification of SOSG fluorescence by the image analysis application Fiji (Schindelin et al., 2012) revealed 2.6× and 3.0× increase in mean fluorescence signal in the biological and artificial low R:FR treatments, respectively, compared with the weed-free control. In addition, mean fluorescence signal in the artificial low R:FR treatments was significantly higher than the biological low R:FR treatment (**Figure 2D**; **Supplementary Table 2**). These results not only indicated that the biological low R:FR treatment could elicit the shade avoidance responses in the absence of direct resource competition but also suggested that the ^1^O_2_, which was predominantly generated by the reflected FR light from neighboring weeds, might be a molecular component of the shade avoidance response.

### Increased leaf production of ^1^O_2_ in the low R:FR light environments is not due to the accumulation of chlorophyll precursors

**Figure 3:**
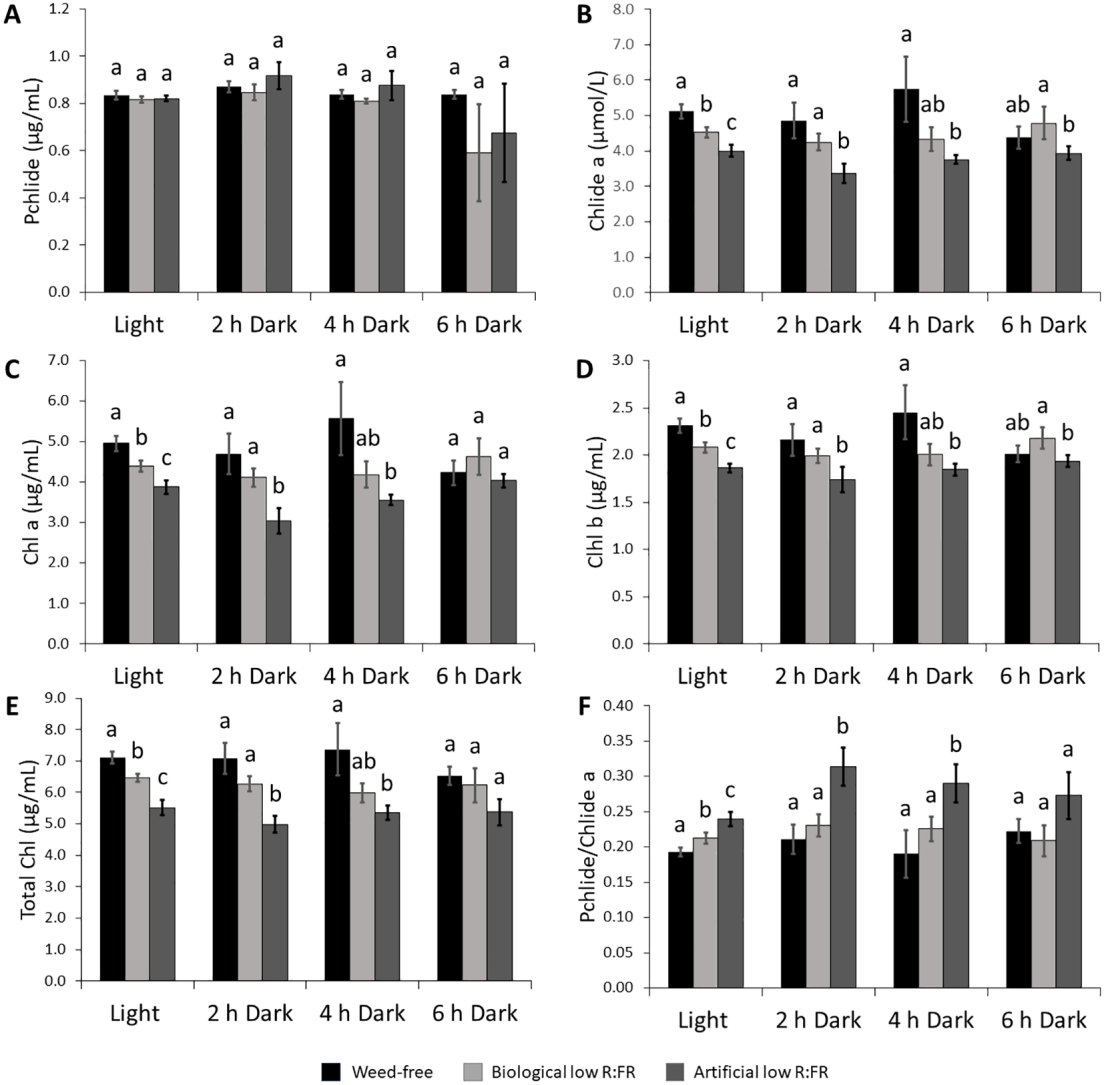
Changes in the levels of photosynthetic pigments in the biological and artificial low R:FR treatments in the light and after two, four, and six hours of dark incubation. *Arabidopsis* plants were exposed to the biological and artificial low R:FR light for 12 hours a day for seven days or kept under control (weed-free) condition prior to sampling. Black bars represent the control while light grey and dark grey bars represent the biological and artificial low R:FR treatments, respectively. No significant change in Pchlide levels in the reflected low R:FR treatments and after dark incubation for two, four, and six hours **(A)**. Decreases in Chlide a levels in the reflected low R:FR treatments and after dark incubation for two and four hours **(B)**. Decreases in Chl a **(C)**, Chl b **(D)**, and total Chl **(E)** levels in the reflected low R:FR treatments and after dark incubation for two and four hours. Increases in the ratio of Pchlide to Chlide a in the artificial low R:FR treatment and after dark incubation for two and four hours **(F)**. Data represent means ± SEM for three independent experiments each consisting of three plants per treatment. Means were separated using Tukey’s HSD test (*P*<0.05). Letters indicate statistical significance of differences across treatments.

Induction of ^1^O_2_ in the *flu* mutant of *Arabidopsis* is the result of photosensitization of accumulated Pchlide following transfer from dark to light (Meskauskiene et al., 2001). To investigate whether a similar mechanism is responsible for increased leaf production of ^1^O_2_ in the low R:FR treatments, the levels of Pchlide, Chlide a, Chl a, Chl b, and total Chl were compared with that of control plants using spectrophotometry. The levels of Pchlide were not significantly altered by the biological and artificial low R:FR treatments (**Figure 3A**), while the Chlide a levels were significantly decreased (9.4% and 25.9%, respectively) compared with the control (**Figure 3B**). In addition, significant decreases were found in the levels of Chl a (10.3% and 8.2%), Chl b (26.9% and 21.9%), and total Chl (9.6% and 25.3%) in the biological and artificial low R:FR treatments compared with the control (**Figures 3C–E**). Further, exposure of *Arabidopsis* plants in the biological and artificial low R:FR treatments to dark periods of two, four, and six hours did not affect Pchlide levels (**Figure 3A**). The Chlide a levels were decreased in the biological and artificial low R:FR treatments (13.9% and 35.7%, respectively) after two hours of dark incubation (**Figure 3B**). This decrease, however, was significant only in the artificial low R:FR treatment. Further, a significant decrease (34%) was found in the artificial low R:FR treatment after four hours of dark incubation. Although the Chlide a levels in the low R:FR treatments were not significantly different from the weed-free control after six hours of dark incubation, the Chlide a level in the artificial low R:FR treatment was significantly lower (19.3%) than the biological low R:FR treatment (**Figure 3B**). The Chl a levels were decreased after two hours (13.5% and 39.4%) and four hours (22.1% and 36.1%) of dark incubation compared with the control (**Figure 3C**). These decreases, however, were significant only in the artificial low R:FR treatment and no significant difference was found between treatments after six hours of dark incubation (**Figure 3C**). The levels of Chl b were decreased after two hours in the biological and artificial low R:FR treatments (9.2% and 24.9%, respectively). This decrease, however, was significant only in the artificial low R:FR treatment. Further, a significant decrease (23.2%) was found in the artificial low R:FR treatment after four hours of dark incubation (**Figure 3D**). Although the levels of Chl b in the biological and artificial low R:FR treatments were not different from the weed-free control after six hours of dark incubation, the level of Chl b in the artificial low R:FR treatment was significantly lower (12.2%) compared with the biological low R:FR treatment (**Figure 3D**). The levels of total Chl were decreased in the biological and artificial low R:FR treatments only after two hours (12.1% and 34.7%) and four hours (20.6% and 31.8%) of dark incubation (**Figure 3E**). Again, these decreases were significant only in the artificial low R:FR treatment (**Figure 3E**). In the biological and artificial low R:FR treatments, *Arabidopsis* plants displayed higher ratios of Pchlide to Chlide a (Pchlide/Chlide a) compared with the control (**Figure 3F**). The higher Pchlide/Chlide a may be due to decreased conversion of Pchlide to Chlide a. It is not clear whether higher Pchlide/Chlide a may contribute to ^1^O_2_ generation. After two and four hours of dark incubation, however, Pchlide/Chlide a were increased in the artificial low R:FR treatment only, whereas six hours of dark incubation did not affect Pchlide/Chlide a (**Figure 3F**). The lack of accumulation of Pchlide in the dark (**Figure 3A**), however, suggests that ^1^O_2_ generation in the low R:FR treatments occurs via a different mechanism than in the *flu* mutant of *Arabidopsis*.

### Low R:FR light environments decrease total carotenoid content and differentially alter levels of xanthophylls

**Figure 4:**
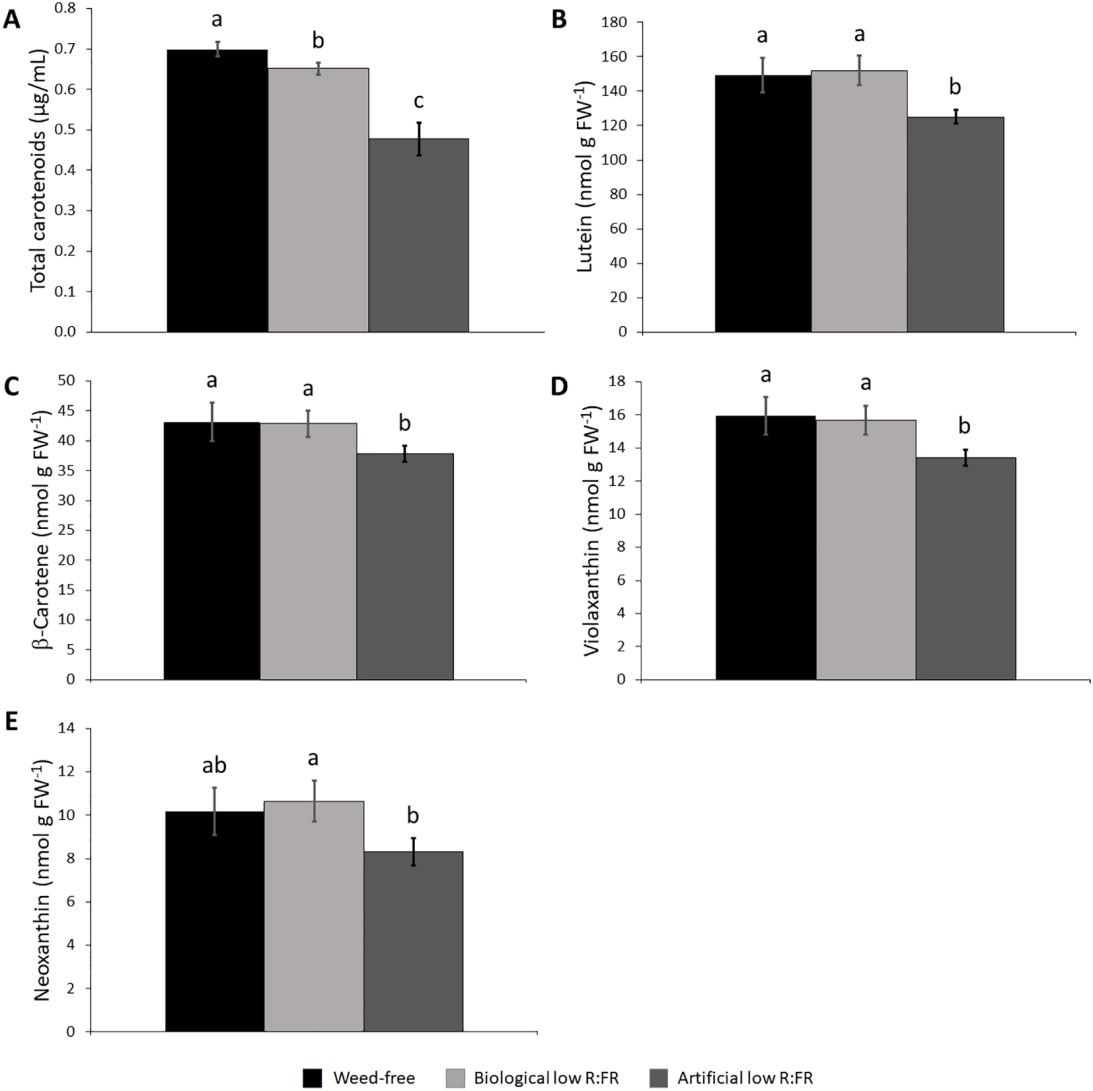
Effect of the reflected low R:FR treatments on carotenoid content. *Arabidopsis* plants were exposed to the biological and artificial low R:FR light for 12 hours a day for seven days or kept under control (weed-free) condition prior to sampling. Black bars represent the control while light grey and dark grey bars represent the biological and artificial low R:FR treatments, respectively. Decreases in total carotenoid content in the biological and artificial low R:FR treatments **(A)** and no changes in the levels of lutein **(B)**, β-carotene **(C)**, violaxanthin, **(D)**, and neoxanthin **(E)** in the biological low R:FR treatment. The levels of lutein, β-carotene, and violaxanthin in the artificial low R:FR treatment are lower than that in the control.Total carotenoid data represent means ± SEM for four replicates consisting of three plants per treatment while individual carotenoid data represent means ± SEM for three independent experiments each consisting of three plants per treatment. Means were separated using Tukey’s HSD test (*P*<0.05). Letters indicate statistical significance of differences across treatments.

Since carotenoids are the most efficient physical quenchers of ^1^O_2_ that primarily protect photosystems from oxidative damage (Triantaphylidès and Havaux, 2009), we investigated whether increased leaf production ^1^O_2_ in the low R:FR light treatments was due to decreases in carotenoid levels. We found that total carotenoid levels were decreased by 7.1% and 37.7% in the biological and artificial low R:FR treatments, respectively (**Figure 4A**). When individual xanthophyll levels were examined by HPLC, however, there appeared to be no difference between the levels of lutein (**Figure 4B**), β-carotene (**Figure 4C**), violaxanthin (**Figure 4D**), and neoxanthin (**Figure 4E**) in the biological low R:FR treatment compared with the control. In the artificial low R:FR treatment, however, the levels of lutein, β-carotene, and violaxanthin were deceased by 17.6%, 13.1%, and 17.1%, respectively, compared with the control. The neoxanthin level in this treatment was significantly lower than in the biological low R:FR treatment but did not differ from that of the control. Therefore, the observed decrease in total carotenoids in the biological low R:FR treatment does not appear to be due to decreases in the levels of lutein, β-carotene, violaxanthin, and neoxanthin. In contrast, the decreased level of total carotenoids in the artificial low R:FR treatment may be attributable to decreases in the levels of lutein, β-carotene, and violaxanthin. Further, these results suggest that the ^1^O_2_ induction by the artificial low R:FR treatment may arise from decreased levels or the suppression of protective function of these carotenoids. These mechanisms however, do not appear to be behind the ^1^O_2_ induction by the biological low R:FR treatment. Unlike the artificial low R:FR treatment, the inability of the biological low R:FR treatment to decrease the levels of the above-mentioned carotenoids may be attributable to the effects associated with the surrogate weed used for the biological low R:FR treatment. We do not, however, rule out the involvement of other carotenoids such as zeaxanthin, which is also a potent physical quencher of ^1^O_2_ (Triantaphylidès and Havaux, 2009).

### Low R:FR light environments increase susceptibility to cell death by ALA

**Figure 5:**
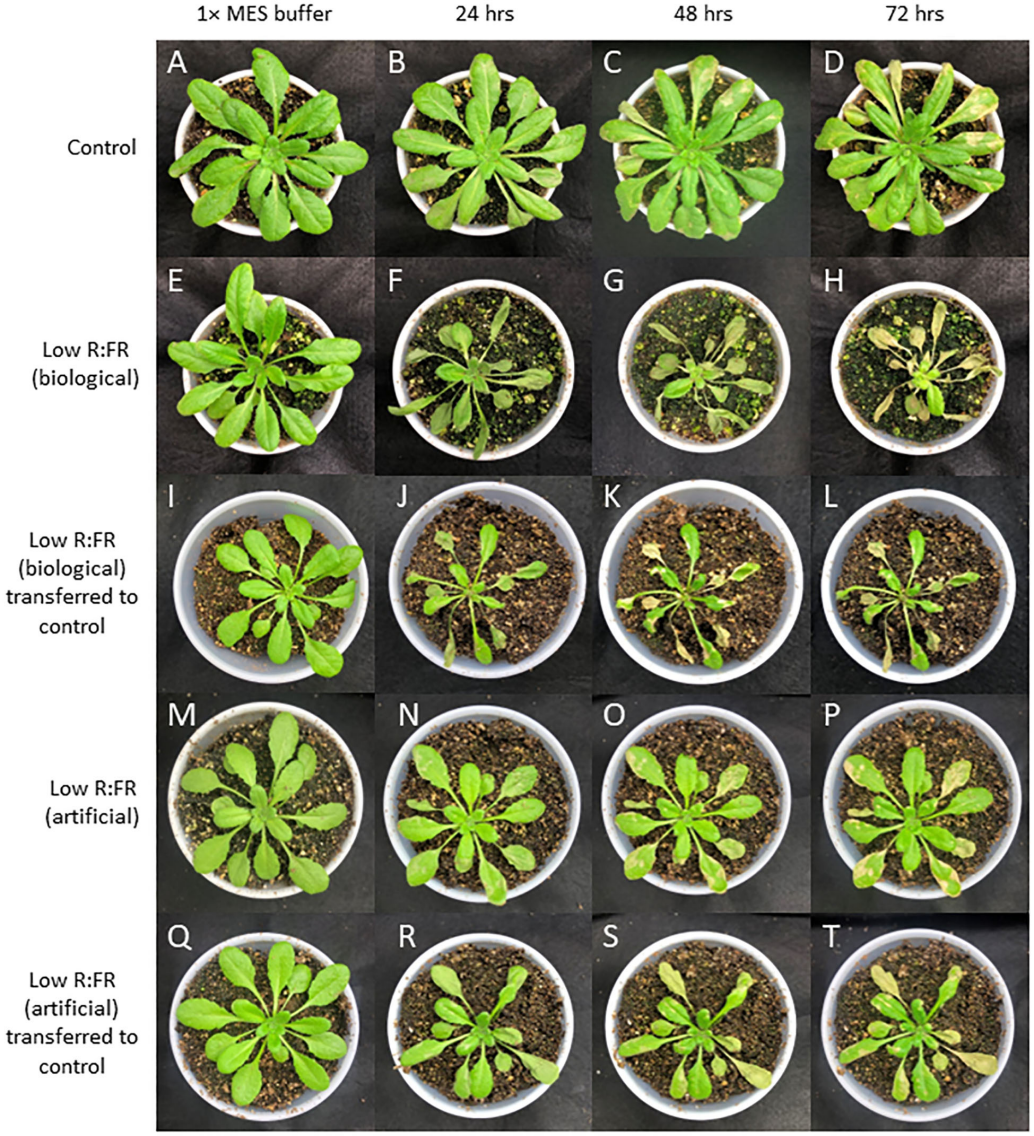
The time course (0-72 h) of *Arabidopsis* responses to ALA in the control, biological and artificial low R:FR treatments. *Arabidopsis* plants were exposed to the biological and artificial low R:FR light for 12 hours a day for seven days or kept under control (weed-free) condition prior to sampling. Control plants for each treatment were sprayed with a two mL solution of 1x MES buffer (10 mM; pH 6.5), while treated plants were sprayed with a two mL solution of 10 mM ALA in 1x MES buffer and placed back in their original treatments or transferred from the reflected low R:FR treatments to the control treatment. Experiments were repeated four times and ALA was sprayed on three plants per treatment per experiment. No damage by 1x MES buffer in the control, but minor damage by ALA after 24 h and localized patches of cell death after 48 and 72 hours **(A–D)**. Signs of wilting 24 h after ALA treatment, progression of cellular damage and necrosis after 24 and 48 hours in the biological low R:FR **(E–H)**. A similar trend after transfer of the ALA-treated plants from the biological low R:FR to the control treatment **(I–L)**. Less severe damage by ALA in the artificial low R:FR light **(M–P)** compared with the biological low R:FR treatment. Persistence of sensitivity to cell death after transfer of the ALA-treated plants from the artificial low R:FR to the control treatment **(Q–T)**.

It is well recognized that both light-dependent and light-independent induction of ^1^O_2_ can trigger cell death responses (Wagner et al., 2004; Ramel et al., 2013a; Mor et al., 2014; Chen and Fluhr, 2018). We sprayed *Arabidopsis* plants in the control, biological, and artificial low R:FR treatments with ALA (10 mM) to increase the levels of photodynamic tetrapyrrole intermediates in these plants and compare their susceptibility to cell death. After 24 hours, plants in the biological low R:FR treatment wilted, while the control displayed little sign of cellular damage (**Figures 5A, B, E, F**). After 48 hours, signs of cellular damage were apparent on all the plants, however, it was more severe in the biological low R:FR treatment (**Figures 5A, C, E, G, M, O**). After 72 hours, the control plants exhibited minor signs of cell death in small localized patches, while in the biological low R:FR treatment, plants displayed total necrosis with the exception of the growing points (**Figures 5A, D, E, H**). Plants in the artificial low R:FR treatment exhibited cell death more severe than the control treatment, but less so than the biological low R:FR treatment (**Figures 5A, D, M, P**). Further, after spraying ALA, plants that were transferred from the low R:FR treatments to the control treatment displayed similar cell death symptoms as the plants that had been replaced into their respective treatments pre-spray (**Figures 5I–L, Q–T**). These results suggest that further increases in ^1^O_2_ levels in the low R:FR treatments may tip the ^1^O_2_ balance from an acclimation response towards a cell death response.

### Low R:FR environments differentially enhance cell death and growth inhibition responses to GSH

**Figure 6:**
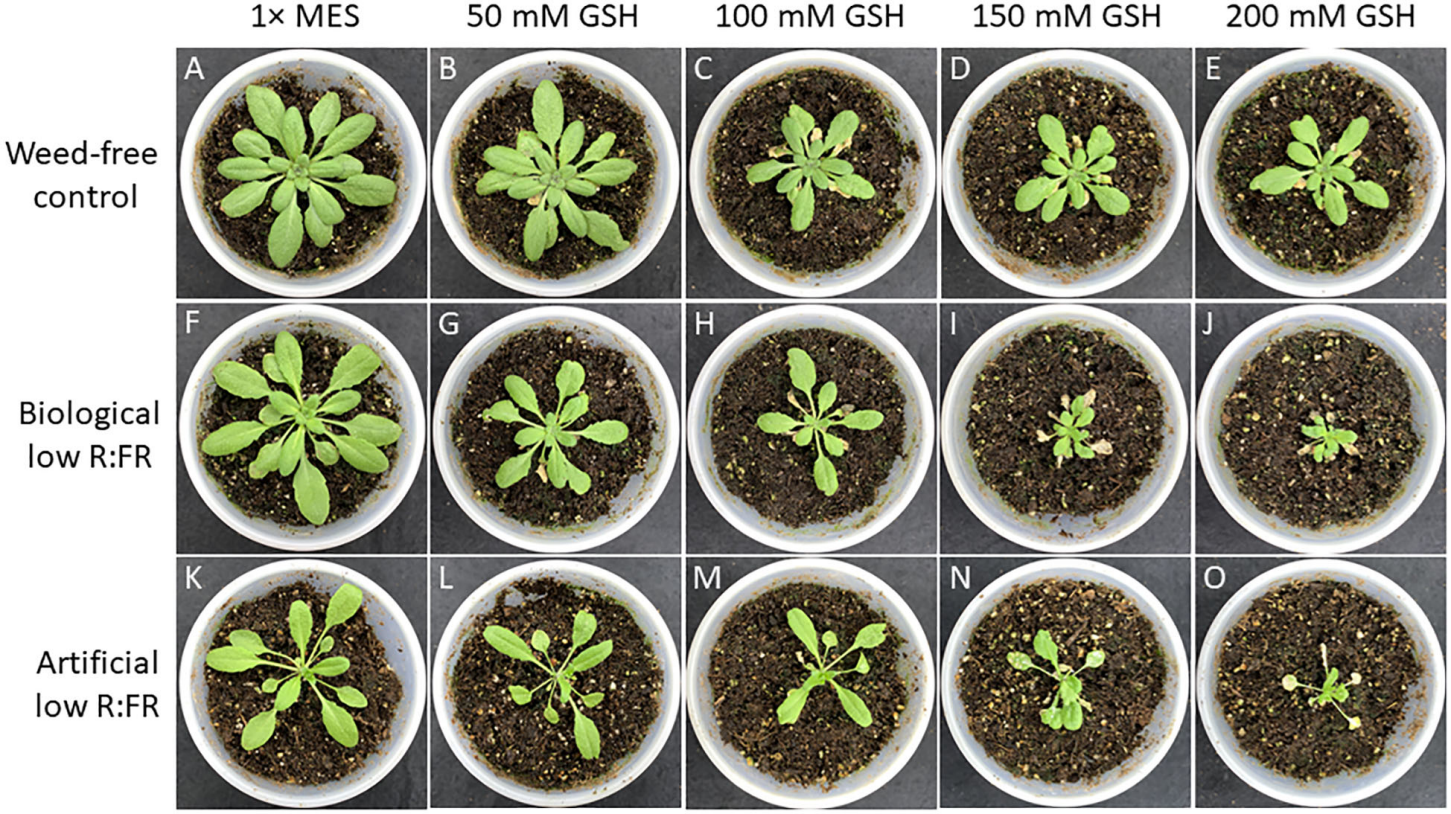
Responses of *Arabidopsis* plants to increasing concentrations of reduced glutathione (GSH) from 50 mM to 200 mM in the weed-free control **(A–E)**, biological low R:FR light **(F–J)**, and artificial low R:FR **(K–O)** light treatments. At the 3-week stage, six *Arabidopsis* plants from each treatment were taken from the growth chambers and all rosette leaves of each plant were sprayed with a one mL solution of each GSH concentration containing 0.01% Tween-20 as a wetting agent. Control plants for each treatment were sprayed with a one mL solution of 1x MES buffer (10 mM, pH 6.5) containing the same concentration of Tween-20. After spraying, the plants were kept outside the growth chambers to dry for one hour and transferred back to the respective treatments. Experiments were repeated three times with five plants per treatment and progression of cell death, and growth inhibition were monitored for one week. Note the dramatic differences in cell death and growth inhibition responses to increasing concentrations of GSH between the control and low R:FR light treatments particularly at 150 mM and 200 mM GSH.

**Figure 7:**
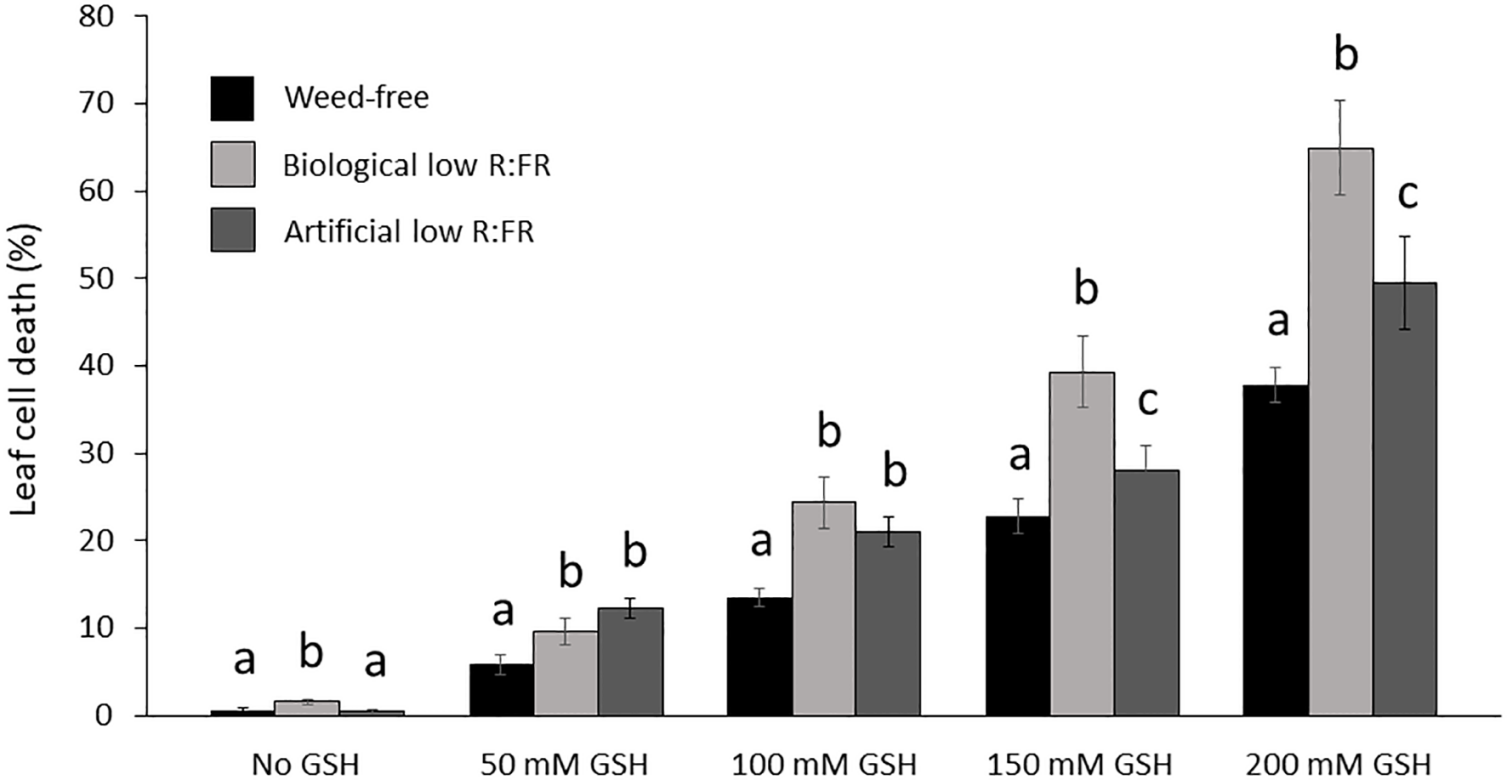
Increases in mean leaf cell death (%) by reduced glutathione (50, 100, 150, and 200 mM GSH) in the weed-free control, biological, and artificial low R:FR light treatments. Plants were treated as described in **Figure 6**. Note the significant increase in mean cell death (%) at each GSH concentration in the low R:FR light treatments relative to the respective weed-free treatment. Data represent means ± SEM for 18 plants per concentration per treatment. Means were separated using Tukey’s HSD test (*P*<0.05). Letters indicate statistical significance of differences across treatments. Images of plants were analyzed by the image analysis application Fiji (Schindelin et al., 2012). For each plant, dead leaf area was determined by subtracting green leaf area from total leaf area and expressed as percentage of leaf cell death (**Supplementary Table 3**).

**Figure 8:**
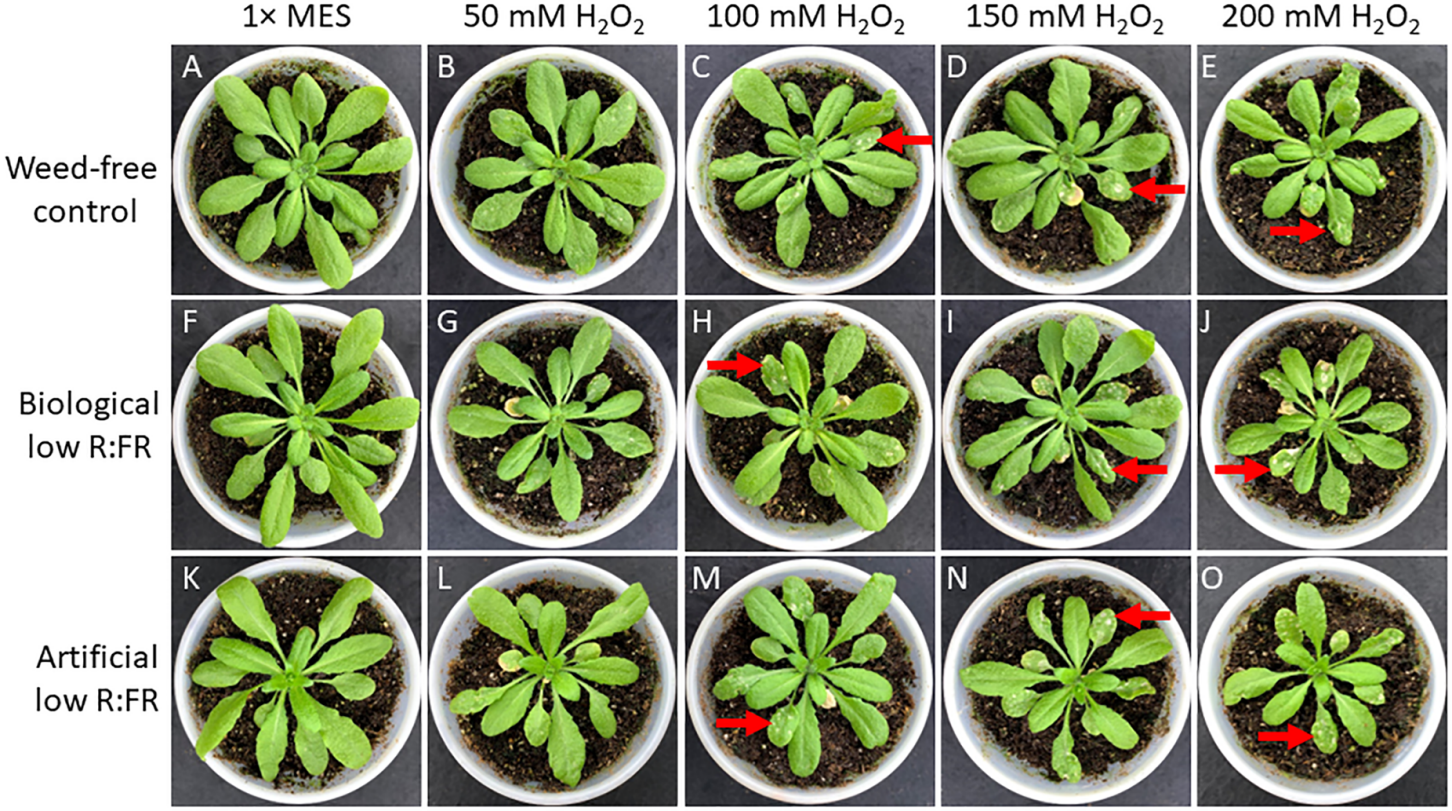
Responses of *Arabidopsis* plants to increasing concentrations of H_2_O_2_ from 50 mM to 200 mM in the weed-free control **(A–E)**, biological low R:FR light **(F–J)**, and artificial low R:FR **(K–O)** light treatments. At the 3-week stage, six *Arabidopsis* plants from each treatment were taken from the growth chambers and all rosette leaves of each plant were sprayed with a one mL solution of each H_2_O_2_ concentration containing 0.01% Tween-20 as a wetting agent. Control plants for each treatment were sprayed with a one mL solution of 1x MES buffer (10 mM, pH 6.5) containing the same concentration of Tween-20. After spraying, the plants were kept outside the growth chambers to dry for one hour and transferred back to the respective treatments. Experiments were repeated three times with five plants per treatment and progression of cell death was monitored for one week. Note that higher concentrations of H_2_O_2_, in contrast to GSH, did not result in a differential cell death in the low R:FR light treatments. Arrows indicate a few patches of cell death across all treatments at higher H_2_O_2_ concentrations.

It has been established that GSH can increase and H_2_O_2_ can decrease photo-oxidative damage through the control of the redox state of the quinone A (Q_A_)-quinone B (Q_B_)-plastoquinone (PQ) pools (Karpinska et al., 2000). We examined susceptibility of *Arabidopsis* plants to GSH and H_2_O_2_ in the low R:FR environments. Treatment of *Arabidopsis* plants with increasing concentrations of GSH (50 mM to 200 mM) resulted in more severe cell death and growth inhibition in the low R:FR environments compared with the weed-free control (**Figure 6**). Quantification of leaf cell death by the image analysis application Fiji (Schindelin et al., 2012) revealed significant increases in leaf cell death (%) in the biological low R:FR (65%, 80%, 73%, and 71%) and artificial low R:FR (110%, 55%, 24%, and 31%) treatments relative to the weed-free treatment at 50, 100, 150, and 200 mM GSH, respectively (**Figure 7**; **Supplementary Table 3**). In contrast, treatment of *Arabidopsis* plants with increasing concentrations of H_2_O_2_ (50 mM to 200 mM) resulted in small and localized patches of cell death in all environments with no distinguishable differences in susceptibility to H_2_O_2_ between the low R:FR light and control environments (**Figure 8**). These results suggest that the effects of GSH on reduction of the Q_A_-Q_B_-PQ pools, efficiency of chloroplast electron transport, and cell death may be exacerbated under low R:FR light environment.

### A few early ^1^O_2_-responsive genes are induced under low R:FR light environments

RNA-sequencing was performed to compare differentially expressed genes (DEGs) in wild type *Arabidopsis* under 12-hour low R:FR light per day for one week and 1931 previously reported early ^1^O_2_-responsive genes in the mutant backgrounds and rose Bengal-treated wild type *Arabidopsis* (op den Camp et al., 2003; Gadjev et al., 2006; Alboresi et al., 2011; Mor et al., 2014). A summary of the number of base pairs sequenced and the number of reads mapped is presented in **Table 1**, which shows an average percentage of mapped reads of 81.7%. Results revealed that 57 of the 1931 ^1^O_2_-responsive genes were differentially expressed in the low R:FR treatments (**Table 2**; **Supplementary Table 4**). Moreover, only six of the 57 ^1^O_2_-responsive genes were commonly up-regulated in the biological and artificial low R:FR treatments, and four previous studies (**Tables 2**, **3**). The minimal similarity between gene expression profiles in the previous four studies and the present study suggests that disparate modes of ^1^O_2_ generation in different genetic backgrounds may elicit unique ^1^O_2_ signatures. Further, growth conditions, tissue types and plant age may also affect ^1^O_2_ signatures. These results also suggest that the levels of ^1^O_2_ in the low R:FR treatments may not be high enough to induce a ^1^O_2_ signaling pathway similar to other *Arabidopsis* systems such as the *flu* mutant (op den Camp et al., 2003), Alternatively, a ^1^O_2_ signaling might have occurred at an earlier time point after the low R:FR light exposure.

**Table 1:** Percentages of sequence reads mapped to the *Arabidopsis thaliana* reference genome.

Treatment	Replicate	No. base pairs sequenced	No. mapped reads	Percent mapped
Weed-free Control	1	65519462	53635248	81.9
	2	53780900	43933391	81.7
	3	98252102	79942744	81.4
Biological low R:FR	1	67826222	55934659	82.5
	2	63146232	51830813	82.1
	3	102906614	84638761	82.2
Artificial low R:FR	1	174670054	141719839	81.1
	2	81621146	66435905	81.4
	3	159953262	130315365	81.5
Average		96408444	78709636	81.7

**Table 2:** Comparison of early ^1^O_2_-responsive DEGs in the reflected low R:FR treatments and four previously studied ^1^O_2_ generating systems.

Category		No. of genes
(1) DEGs in four plant ^1^O_2_ generating systems^a^		1931
(2) Common DEGs identified in (1) and biological low R:FR		57
(3) Common DEGs identified in (1), (2) and artificial low R:FR		6
Up (+) or down (-) regulated in (1)	Up (+) or down (-) regulated in (2)	No. of genes
**+**	**+**	21
**-**	**-**	5
**+**	**-**	9
**-**	**+**	17

**^a^** op den Camp et al., 2003; Gadjev et al., 2006; Alboresi et al., 2011; Mor et al., 2014.

*Arabidopsis* plants were exposed to the biological and artificial low R:FR light for 12 hours a day for seven days or kept under control (weed-free) condition prior to sampling. Six ^1^O_2_-responsive genes were consistently upregulated in the reflected low R:FR treatments and other four ^1^O_2_ generating systems (op den Camp et al., 2003; Gadjev et al., 2006; Alboresi et al., 2011; Mor et al., 2014). Data represent three independent experiments with a total of nine samples from three treatments (weed-free, biological low R:FR, and artificial low R:FR light) with three replicates, each consisting of a pool of leaves from three individual plants.

**Table 3:** Induction of early ^1^O_2_-responsive and sulfotransferase genes by the biological and artificial low R:FR light treatments.

DEGs	Gene name/TAIR description	Log_2_FC (biological)	Log_2_FC (artificial)	*P*-value
	Early ^1^O_2_ responsive genes			
(1) *At3g10720*	*INV*; pectin methylesterase inhibitor	1.18	0.24	3.5 x10^-3^
(2) *At3g19380*	*PUB25*; U-box E3 ubiquitin ligase	0.75	0.34	2.2 x10^-3^
(3) *At4g01870*	*TolB*; tolB protein-like protein	0.73	0.02	1.6 x10^-2^
(4) *At5g57560*	*TCH4*; a cell wall-modifying enzyme	1.64	0.07	1.9 x10^-2^
(5) *At1g44350*	*ILL6*; IAA-leucine resistance (ILR)-like6	3.26	0.09	2.6 x10^-3^
(6) *At5g24810*	*ABC1K11*; ABC1 family protein	0.97	0.09	1.2 x10^-2^
	Sulfotransferases			
(7) *At5g07010*	*ST2a*; sulfotransferase 2a	3.32	10.02	7.7 x10^-9^
(8) *At5g07000*	*ST2b*; sulfotransferase 2b	1.16	2.23	4.9 x10^-3^

*Arabidopsis* plants were exposed to the biological and artificial low R:FR light for 12 hours a day for seven days or kept under control (weed-free) condition prior to sampling. Data represent three independent experiments with a total of nine samples from three treatments (weed-free, biological low R:FR, and artificial low R:FR light) with three replicates, each consisting of a pool of leaves from three individual plants.

### Negative regulators of jasmonate accumulation are induced under low R:FR light environments

Increases in ^1^O_2_ levels in the low R:FR treatments were below a threshold to induce a cell death response (**Figures 1**, **2**). The lack of a cell death response suggested induction of a ^1^O_2_ acclimation response, which is known to be induced by low levels of JA (Ramel et al., 2013b). Therefore, we sought to determine whether genes involved in regulation of bioactive JA levels were up-regulated by the low R:FR treatments. RNA-seq analysis revealed that the early ^1^O_2_-responsive gene *ILL6* (op den Camp et al., 2003), which is involved in negative regulation of bioactive JA levels (Bhosale et al., 2013), was up-regulated (**Table 3**). We also found that not only the sulfotransferase *ST2a* but also the closely related *ST2b* was up-regulated in the biological and artificial low R:FR treatments (**Table 3**). Up-regulation of the sulfotransferase *ST2a* that catalyzes the conversion of 12-hydroxy JA (OH-JA) to JA sulfate (HSO_4_-JA) (Gidda et al., 2003) is a major mechanism diverting JA precursors from bioactive JA pools thus attenuating JA signaling under low R:FR light (Fernández-Milmanda et al., 2020). Since *ST2b* lacks sulfotransferase activity (Gidda et al., 2003; Fernández-Milmanda et al., 2020), its function under low R:FR treatments is not clear. The relationships between JA accumulation and ^1^O_2_-induced cell death (Przybyla et al., 2008), and between suppression of JA synthesis and photo-tolerance (Ramel et al., 2013a, b) have been well established. Therefore, the up-regulation of *ILL6* and *ST2a* in the low R:FR treatments may represent a possible connection between the low R:FR light, attenuation of bioactive JA levels, and ^1^O_2_ acclimation response.

### Induction of jasmonate repressors connects reflected far-red light cues from neighboring weeds with ^1^O_2_ acclimation response

**Figure 9:**
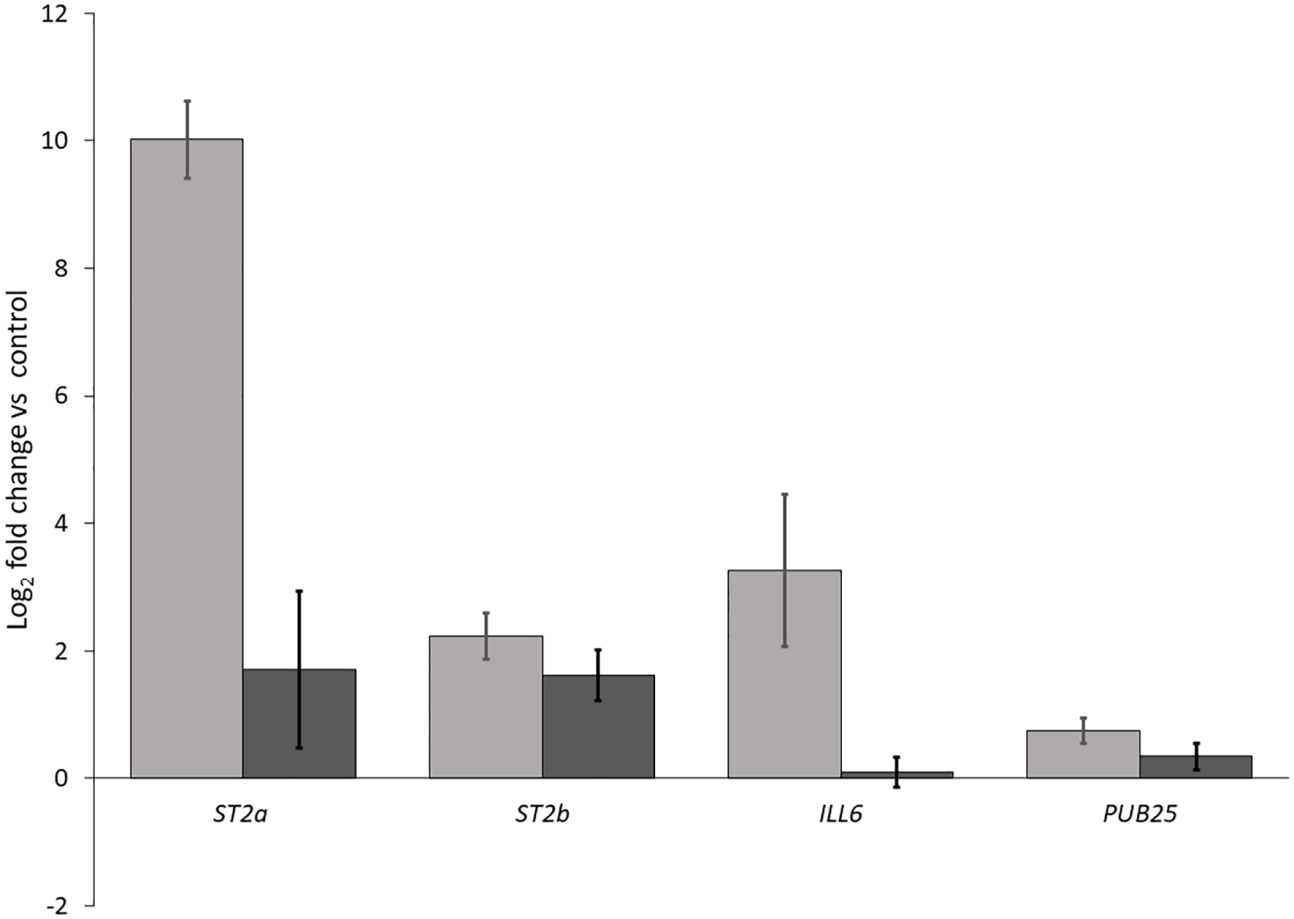
Increases in transcript levels of *ST2a*, *ST2b*, *ILL6*, and *PUB25* in the biological low R:FR treatment relative to the weed-free control. Data represents fold change gene expression in the biological low R:FR light relative to the weed-free control treatment ± SEM for two independent experiments each consisting of leaves from four plants per treatment (*P* ≤ 0.05).

We performed two independent RT-qPCR assays to investigate whether the reflected far-red light from neighboring weeds was indeed responsible for the induction of JA repressors and ^1^O_2_ responsive genes. We included in our experiments *ST2a* (a negative regulator of JA) and the closely related *ST2b*, as well as the early ^1^O_2_ responsive genes *ILL6*, which is also a negative regulator of JA, and *PUB25*, which encodes a U-box E3 ligase involved in plant organ growth. We found that all of these transcripts in *Arabidopsis* leaves were at higher levels in the biological low R:FR treatment relative to weed-free control (**Figure 9**) confirming the expression profile of the selected genes in RNA-seq results (**Table 3**). These results further suggested that hydrolysis of bioactive JA conjugates (JA-isoleucine) by the ^1^O_2_ responsive *ILL6* and sulfation of JA metabolites by *ST2a* may act to promote both the shade avoidance response and the ^1^O_2_ acclimation response under resource-independent weed competition.

## Discussion

Our results indicate that induction of SAR in *Arabidopsis* due to proximity to neighboring weeds coincides with increased leaf production of ^1^O_2_ (**Figures 1**, **2**). Some of the elongation responses are modulated by the low R:FR light-mediated elevation of the volatile hormone ethylene in plants or in the canopy environment (Pierik et al., 2004a, b). Other reports have shown the suppression of terpenoids and other green leaf volatiles under low R:FR light (Kegge et al., 2013) and suggested the dominant effect of light signaling over volatile cues during weed-crop interactions (Pierik and de Wit, 2014). We are not, however, aware of any previous work describing ^1^O_2_ induction in plants by volatile compounds emanating from neighboring weeds. Moreover, a similar ^1^O_2_ increase along with induction of the SAR in the artificial low R:FR treatment (**Figures 1**, **2**) suggests that the low R:FR light emanating from neighboring weeds may be the main signal responsible for increased ^1^O_2_ production.

Environmental stress factors elevate reactive oxygen species (ROS) levels in plants (Fryer et al., 2002; Hideg et al., 2002; Xiong et al., 2002; Apel and Hirt, 2004). Disparate ROS such as ^1^O_2_ and O_2_^-^/H_2_O_2_, which are elevated under disparate physiological conditions (Apel and Hirt, 2004), may also exhibit antagonistic interactions under certain conditions. This antagonistic interaction was exemplified in the *flu* mutant of *Arabidopsis* in which suppression of H_2_O_2_ by overexpression of a thylakoid-bound ascorbate peroxidase resulted in enhanced ^1^O_2_-mediated cell death and growth inhibition (Laloi et al., 2007). Also, the simultaneous induction of ^1^O_2_ and reduction of O_2_^-^ in the etiolated *phytochrome interacting factor 3* and *phytochrome interacting factor quadruple* mutants of *Arabidopsis* upon transfer to light has been attributed to the antagonistic effect of ^1^O_2_ on O_2_^-^ and H_2_O_2_ (Chen et al., 2013). Given the established specificity of ^1^O_2_- and O_2_^-^/H_2_O_2_-dependent signaling in the *flu* mutant of *Arabidopsis* (Laloi et al., 2007), the ^1^O_2_ induction in the low R:FR light treatments may give rise to a distinct stress signaling response. A recent study found no increase in the intensity of SOSG fluorescence in *Arabidopsis* leaf discs following a two-hour exposure to supplemental FR light (Dmitrieva et al., 2021). This discrepancy may be due to small size of leaf discs (0.5 × 0.5 cm), lower concentration of SOSG (5 µM), and the short duration of exposure to FR light (two hours). Several lines of evidence indicate that a FR light pre-treatment of *Arabidopsis* results in Pchlide accumulation (Sperling et al., 1997; McCormac and Terry, 2002). Further, photo-excitation of Pchlide following transfer of *Arabidopsis* from FR light to white light resulted in rapid generation of ^1^O_2_ (Page et al., 2017). In the absence of FR light, Pchlide accumulation in the dark and photo-excitation following transfer to light is the mechanism behind rapid ^1^O_2_ generation in the *flu* mutant (op den Camp et al., 2003). Also, differential effects of several components of the *Arabidopsis* light signaling pathway (PHYTOCHROMES, PHYTOCHROME INTERACTING FACTORS, ELONGATED HYPOCOTYL 5, ELONGATED HYPOCOTYL 5 HOMOLOG, and CONSTITUTIVE PHOTOMORPHOGENIC 1) on ^1^O_2_ production and cell death following transfer to light corresponded closely to the Pchlide levels formed during seedling de-etiolation (Chen et al., 2013). Accumulation of Pchlide, however, does not appear to be the main cause of ^1^O_2_ generation in the wild-type *Arabidopsis* under the low R:FR light treatments as Pchlide levels were not significantly different from the control, nor did Pchlide levels increase following dark incubations for up to six hours (**Figure 3A**). Although the mechanism of ^1^O_2_ generation under our low R:FR treatments is not clear, decreased levels of Chlide a (**Figure 3B**), and therefore, increases in Pchlide to Chlide ratios (**Figure 3F**) may be interpreted as slower conversion rates of Pchlide to Chlide allowing for photo-excitation of a part of free Pchlide and thus ^1^O_2_ generation.

Carotenoids (car) including lutein, zeaxanthin, and β-carotene are potent physical and chemical quenchers of ^1^O_2_ (Trebst, 2003; Triantaphylidès and Havaux, 2009; Dogra and Kim, 2020). The lack of zeaxanthin and lutein in the lycopene-ε-cyclase and violaxanthin de-epoxidase double mutant (*npq1 lut2*) of *Arabidopsis* resulted in ^1^O_2_ generation under a combination of low temperature and high light stress (Alboresi et al., 2011). In addition, ^1^O_2_ generation in the *npq1 lut2* mutant was accompanied by an increase in Chl a/Chl b and a decrease in Chl/Car reflecting the reduction of PSII/PSI and induction of carotenoid biosynthetic genes as ^1^O_2_-mediated acclimation responses (Alboresi et al., 2011). Under the low R:FR treatments, however, ^1^O_2_ induction did not coincide with such increases (data not shown), indicating differences in ^1^O_2_ acclimation responses between these ^1^O_2_ generating systems under two different stresses. In addition, the levels of lutein, β-carotene, violaxanthin, and neoxanthin were not affected by the biological low R:FR treatment, while total carotenoid levels were decreased in the biological and artificial low R:FR treatments (**Figure 4**). Decreases in chlorophylls and carotenoids are among universal responses to low R:FR light (Meng et al., 2019; Zhen and Bugbee, 2020; Kong and Nemali, 2021; Frosch and Mohr, 1980; Li and Kubota, 2009) and are regulated by phytochrome in a coordinated and co-localized manner (Meier et al., 2011; Rodríguez-Villalón et al., 2009; Welsch et al., 2000; Von Lintig et al., 1997). Although carotenoid deficiency may lead to ^1^O_2_ generation (Krieger-Liszkay, 2005), it is not clear whether decreases in chlorophylls (**Figure 3**) and total carotenoids (**Figure 4**) in the low R:FR treatments contribute to ^1^O_2_ generation.

It is known that ALA is the first committed compound in the synthesis of tetrapyrrole pigments (Tanaka and Tanaka, 2007; Beale, 1990). Earlier studies have shown that plants fed with exogenous ALA accumulate tetrapyrrole intermediates including protoporphyrin IX and Pchlide (Gough, 1972; Mascia, 1978). These intermediates are highly photodynamic and generate ^1^O_2_ as the major ROS in the light (Becerril and Duke, 1989; op den Camp et al., 2003; Shao et al., 2007; Rebeiz et al., 1988). Similarly, exogenous treatment of *Arabidopsis* leaves with ALA resulted in a cell death response, which was exaggerated under the low R:FR treatments (**Figure 5**). This differential response cannot be attributed to higher Pchlide level prior to ALA feeding as Pchlide levels in the low R:FR treatments were not significantly different from the control (**Figure 3A**). Given the direct relationship between the ^1^O_2_ levels and the extent of cell death in *Arabidopsis* leaves (op den Camp et al., 2003; Laloi et al., 2007), the differential cell death response under the low R:FR treatments (**Figure 5**) may be due to higher ^1^O_2_ levels prior to the ALA treatment. This may increase susceptibility to cell death following ALA feeding. Such increased susceptibility to cell death due to higher steady-state cellular levels of ROS has been shown in tobacco plants treated with the signaling molecules salicylic acid and nitric oxide (Amirsadeghi et al., 2006). This ^1^O_2_-mediated susceptibility to cell death by ALA may provide an explanation for the differential cell death responses of *Arabidopsis* plants under low R:FR light treated with ALA and transferred to the control (**Figures 5I–L, Q–T**). This, to some extent, is reminiscent of the increased sensitivity to oxidative stress and cell death due to a salicylic acid-dependent accumulation of O_2_^-^ and H_2_O_2_ under short days in the *fhy3 far1* double mutant of *Arabidopsis* lacking two light signaling components FAR-RED ELONGATED HYPOCOTYL3 (FHY3) and FAR-RED IMPAIRED RESPONSE1 (FAR1) (Ma et al., 2016). On the other hand, the cell death response in the *AtIPS1* mutant of *Arabidopsis* lacking 1L-myo-inositol-1-phosphate synthase activity under long days did not result in increased ROS sensitivity (Meng et al., 2009). Therefore, we do not exclude the possibility that factors other than higher steady state ^1^O_2_ levels in the low R:FR treatments may contribute to increased susceptibility to cell death by ALA.

A recent study indicated that the complexity of leaf tissue including the cuticle layer shielding the upper and lower epidermis could limit SOSG penetration into *Arabidopsis* leaves (Prasad et al., 2018). Force infiltration with a syringe led to nonuniform penetration and distribution of SOSG in the leaf spaces while the resulting mechanical injury induced a strong SOSG fluorescence signal (Prasad et al., 2018). In contrast, pressure infiltration of *Arabidopsis* leaf pieces with a shut syringe resulted in uniform delivery of SOSG to the leaf tissue without ^1^O_2_ induction (Prasad et al., 2018). In our experiments, we used pressure infiltration with a 60 mL syringe allowing for complete infiltration of whole plant tissue while avoiding mechanical injury that could trigger ^1^O_2_ production. Given the complexity of leaf tissue and the uneven occurrence of SOSG fluorescence signal in the leaf samples (**Figures 2B, C**), we do not rule out the possibility of nonuniform generation of SOSG in the leaf cell compartments such as chloroplasts in the cells across the leaf tissue. This uneven occurrence of SOSG fluorescence signal is to some extent similar to that in *Arabidopsis* leaves following transfer from FR light to white light (Page et al., 2017). In addition, increases in mean fluorescence signal in the biological low R:FR (2.6×) and artificial low R:FR (3.0×) (**Figure 2D**) were similar to that reported one hour after the transfer of FR light-treated *Arabidopsis* to white light (Page et al., 2017).

High concentrations of H_2_O_2_ and GSH in the chloroplast can increase the degree of oxidation and reduction of quinone A (Q_A_), respectively. While higher degrees of Q_A_ oxidation by H_2_O_2_ enhance the efficiency of electron transport in PS II, higher degrees of Q_A_ reduction by GSH have the opposite effect. Therefore, the H_2_O_2_ action can decrease and the GSH action can increase the extent of photo-oxidative stress and photoinhibition (Karpinska et al., 2000). Based on the opposing effects of H_2_O_2_ and GSH on the redox state of Q_A_ and efficiency of electron transport in PS II, it was hypothesized that treatment of the *flu* mutant of *Arbabidopsis* with H_2_O_2_ may reduce and treatment with GSH may increase ^1^O_2_ production and growth inhibition (Laloi et al., 2007). Given that our experimental low R:FR environments enhanced ^1^O_2_ production, we examined whether GSH treatment could enhance cell death. Although it was not clear to what extent GSH could reach the chloroplasts, our observations indicated that higher concentrations of GSH could result in differential cell death and growth inhibition responses in the low R:FR environments (**Figures 6**, **7**). This was, however, not the case in the H_2_O_2_-treated plants. Increasing concentrations of H_2_O_2_ resulted in a few localized patches of cell death in all treatments (**Figure 8**). The development of these patches of cell death in all treatments may be due to inability of the H_2_O_2_ detoxifying systems to scavenge excess H_2_O_2_ at higher concentrations. While in some cases, FR light supplementation has enhanced photosynthetic efficiency of shorter wavelength light (Zhen and van Iersel, 2017), in other cases it has led to overproduction of ROS and damage to PSII and PSI (Tjus et al., 2001). Preferential excitation of PSI over PSII by FR light would enhance electron transfer from the PQ pools to PSI, which would be supplied by GSH. This would mitigate overreduction of PQ pools. Therefore, the observed increases in cell death by GHS in the low R:FR treatments (**Figure 6**; **Supplementary Table 3**) may be due to factors other than over-reduction of PQ pools. Alternatively, the damage to PSI and to a greater extent to PSII by FR light-induced ROS (Tjus et al., 2001) would affect the efficiency of electron transport, in which case, addition of excess electron to PQ pools via GSH may exacerbate ROS damage leading to increased cell death.

Our results suggest that under reflected low R:FR light, wild type *Arabidopsis* generates ^1^O_2_ via a mechanism different from those reported in four plant ^1^O_2_ generating systems (**Table 2**), allowing the identification of the transcriptomic signature of ^1^O_2_ signaling. Differential expression of only 3.2% of the previously reported early ^1^O_2_-responsive genes (57 out of 1931) suggests specificity of the ^1^O_2_ signaling under reflected low R:FR light stress (**Table 2**; **Supplementary Table 4**). This specificity is further highlighted by the up-regulation of only six previously reported ^1^O_2_-responsive genes in the biological and artificial low R:FR light treatments (**Table 3**). Among these, *ILL6*, which is also known to be rapidly induced within two hours following ^1^O_2_ release (op den Camp et al., 2003), encodes an amidohydrolase that catalyzes the cleavage of isoleucine (Ile) from JA-Ile thus making JA biologically inactive (Bhosale et al., 2013). Recently, the up-regulation of *ILL6* was reported under low R:FR light as part of a mechanism that attenuates the JA signaling pathway (Fernández-Milmanda et al., 2020). Our results suggest that the up-regulation of *ILL6* in the reflected low R:FR treatments may be an ^1^O_2_ response, which raises the intriguing possibility that attenuation of the JA signaling pathway may be partly mediated via a ^1^O_2_ signaling pathway. This attenuation is a major process that generates a trade-off between defense and growth in favor of the SAR under plant competition (Fernández-Milmanda et al., 2020). It has been demonstrated that attenuation of the JA signaling pathway under a 10-hour low R:FR light treatment occurs via the up-regulation of the sulfotransferase *ST2a.* The *ST2a* encoded protein exclusively catalyzes the conversion of JA to sulfated JA (HSO_4_-JA), while the closely related *ST2b* fails to respond to the above FR light treatment and is dispensable for the accumulation of HSO_4_-JA (Fernández-Milmanda et al., 2020). We found that both *ST2a* and *ST2b* were up-regulated under a 12-hour reflected low R:FR light treatment for seven days (**Table 3**; **Figure 9**) and cannot exclude the possibility that *ST2a* and *ST2b* may be ^1^O_2_-responsive. The discrepancy in the *ST2b* response may be due to differences in experimental FR light conditions.

Upregulation of *ST2a* and *ST2b* have been shown in a transgenic *Arabidopsis* line with suppressed mitochondrial serine acetyltransferase (SAT3) level (Haas et al., 2008). It is noteworthy that upregulation of *ST2a* and *ST2b* in the SAT3 line coincided with the downregulation of disease or pathogen response genes whose responses are coordinated by methyl jasmonate (Haas et al., 2008). Although the upregulation of *ST2b* (**Table 3**; **Figure 9**) suggests that it may play a role under certain low R:FR light conditions, it is not feasible to predict the function of a plant sulfotransferase solely based on its sequence similarity to sulfotransferases with known functions (Hirschmann et al., 2014). So far, there is no evidence for the involvement of *ST2b* in the JA pathway. Initial screens for *ST2b* substrates found no activity against hydroxyjasmonate compounds (Gidda et al., 2003). Further, a recent study found no evidence for the JA sulfation by *ST2b* under low R:FR light (Fernández-Milmanda et al., 2020).

An important question regarding ^1^O_2_ induction under reflected low R:FR light is whether ^1^O_2_ serves a physiological function under plant competition. Under this condition, generation of high levels of ^1^O_2_ can place the rapidly growing plants at a disadvantage as high levels of ^1^O_2_ can steer the cell fate towards cell death (Danon et al., 2005; Kim et al., 2012). We envision a possibility that the low R:FR-mediated attenuation of JA accumulation by *ST2a* and *ILL6* may result in an acclimation response to ^1^O_2_. This acclimation response, in contrast to a cell death response (Ramel et al., 2013b), may have a growth inhibitory effect similar to that in the *flu* mutant (Laloi et al., 2006, 2007). This growth inhibitory effect of ^1^O_2_ may be exploited by plants under competition to modulate elongation growth to avoid excess elongation in anticipation of oncoming competition. The growth inhibitory effect of ^1^O_2_ may also be exerted via the action of other ^1^O_2_-responsive genes. In this regard, overexpression of the early ^1^O_2_-responsive *PUB25*, which encodes a U-box E3 ubiquitin ligase (**Table 3**), is known to cause growth inhibition in *Arabidopsis* (Li et al., 2021). Interestingly, *PUB25* was among the ^1^O_2_-responsive genes that were upregulated under the biological and artificial low R:FR treatments (**Table 3**), and may play a role in modulation of growth by ^1^O_2_.

In closing, our results indicate that ^1^O_2_ production is an early response to reflected FR light from neighboring weeds. Our biological low R:FR light treatment allows the investigation of ^1^O_2_ acclimation responses under resource-independent competition. Under this condition, ^1^O_2_ itself may contribute to the acclimation response by up-regulating genes such as *ILL6*, which acts to reduce the pool of bioactive JAs. Further, the demonstration of up-regulation of *ST2b* under artificial and biological low R:FR light environments allows further investigations into the role of *ST2b* in plant competition.

## Data Availability

The datasets presented in this study can be found in online repositories. The names of the repository/repositories and accession number(s) can be found in the article/**Supplementary Material**.

## Supplementary material

Supplementary Table 1 Incoming and reflected light spectral composition in the weed-free control, biological low R:FR and artificial low R:FR treatments.

Supplementary Table 2 Fluorescence intensity in leaf images of SOSG-treated *Arabidopsis* plants in the weed-free control, biological low R:FR and artificial low R:FR treatments.

Supplementary Table 3 Image analysis of leaf cell death in GSH-treated *Arabidopsis* plants in the weed-free control, biological low R:FR and artificial low R:FR treatments.

Supplementary Table 4 Expression profiles of ^1^O_2_-responsive genes in the biological and artificial low R:FR treatments.
