# Supplementary material for: Plant competition cues activate a singlet oxygen signaling pathway in Arabidopsis thaliana
Source: Front Plant Sci. 2024 Aug 20;15:964476. doi: 10.3389/fpls.2024.964476 (PMC11368760; doi:10.3389/fpls.2024.964476)
Supplement: Supplementary file 5 [file Presentation1.pptx]

## Slide 1
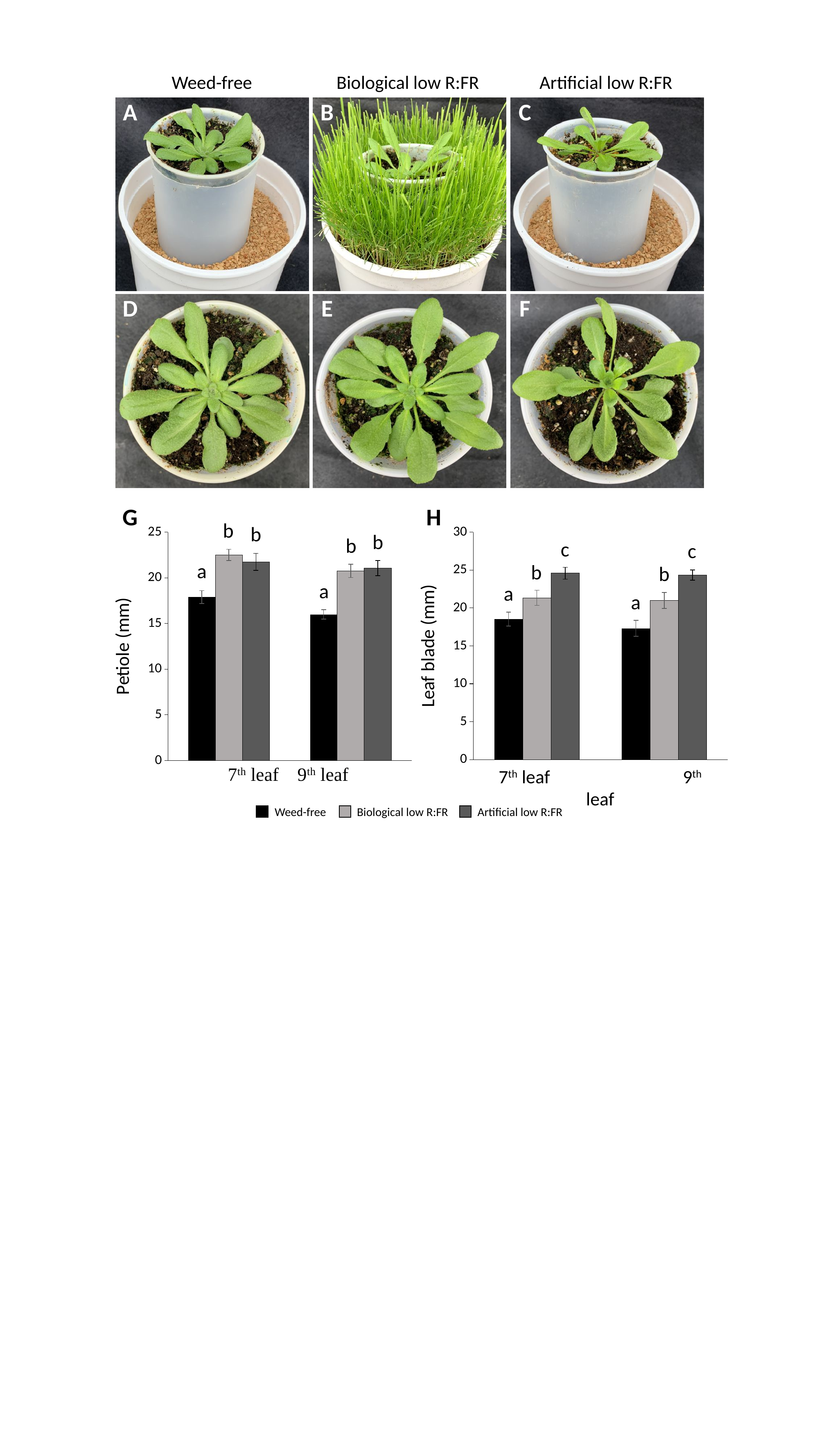

Weed-free
Biological low R:FR
Artificial low R:FR
A
B
C
D
E
F
H
G
b
### Chart
| Category | | | |
|---|---|---|---|
| 7th leaf | 17.886 | 22.502999999999997 | 21.737000000000002 |
| 9th leaf | 15.99111111111111 | 20.750999999999998 | 21.059999999999995 |
### Chart
| Category | | | |
|---|---|---|---|
| 7th leaf | 18.535999999999998 | 21.338 | 24.58 |
| 9th leaf | 17.31555555555555 | 20.985999999999997 | 24.343 |b
b
b
c
c
a
b
b
a
a
a
7th leaf			9th leaf
Weed-free
Biological low R:FR
Artificial low R:FR
