# Supplementary material for: Plant competition cues activate a singlet oxygen signaling pathway in Arabidopsis thaliana
Source: Front Plant Sci. 2024 Aug 20;15:964476. doi: 10.3389/fpls.2024.964476 (PMC11368760; doi:10.3389/fpls.2024.964476)
Supplement: Supplementary file 6 [file Presentation2.pptx]

## Slide 1
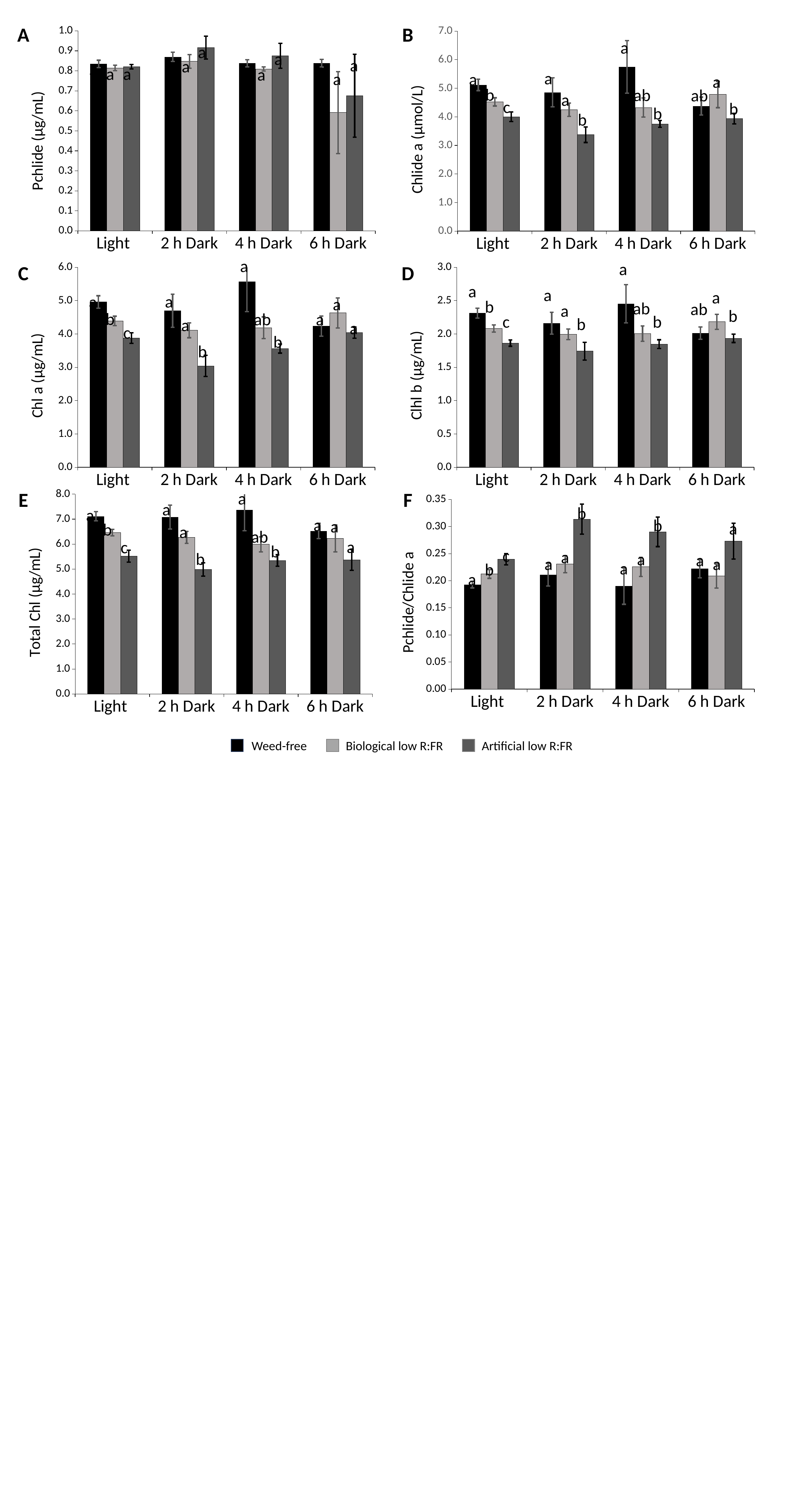

B
A
### Chart
| Category | | | |
|---|---|---|---|
| Light | 0.8348600000000003 | 0.814417777777778 | 0.8207311111111112 |
| 2 h Dark | 0.8697911111111114 | 0.8473511111111114 | 0.915977777777778 |
| 4 h Dark | 0.8379555555555558 | 0.8090777777777781 | 0.8757000000000001 |
| 6 h Dark | 0.838168888888889 | 0.5915755555555555 | 0.6755644444444446 |
### Chart
| Category | | | |
|---|---|---|---|
| Light | 5.115971666666667 | 4.524038333333333 | 4.003116666666667 |
| 2 h Dark | 4.857546666666667 | 4.2513716666666665 | 3.3727950000000004 |
| 4 h Dark | 5.749951666666667 | 4.326063333333333 | 3.752978 |
| 6 h Dark | 4.378498333333333 | 4.782150000000001 | 3.9376900000000012 |b
a
c
b
b
### Chart
| Category | | | |
|---|---|---|---|
| Light | 4.95995 | 4.390585000000001 | 3.874805 |
| 2 h Dark | 4.6965933333333325 | 4.109895 | 3.040703333333333 |
| 4 h Dark | 5.570081666666667 | 4.183821666666666 | 3.5596159999999997 |
| 6 h Dark | 4.231749999999999 | 4.62935 | 4.039255 |
### Chart
| Category | | | |
|---|---|---|---|
| Light | 2.3124199999999995 | 2.0817683333333337 | 1.8620783333333335 |
| 2 h Dark | 2.1610333333333336 | 1.9936333333333334 | 1.7411033333333332 |
| 4 h Dark | 2.452953333333333 | 2.005285 | 1.8471680000000004 |
| 6 h Dark | 2.0126616666666663 | 2.181153333333333 | 1.9343599999999999 |D
C
b
b
c
b
b
c
b
b
F
E
### Chart
| Category | | | |
|---|---|---|---|
| Light | 0.1928812018753114 | 0.2125174839819979 | 0.23963270657015465 |
| 2 h Dark | 0.21098817273994672 | 0.23078851509704942 | 0.3136588955546294 |
| 4 h Dark | 0.19003323900452848 | 0.2255214186753427 | 0.29018873543756624 |
| 6 h Dark | 0.2223097279750839 | 0.20872528885908023 | 0.27303273875564166 |b
b
b
c
b
c
b
Weed-free
Biological low R:FR
Artificial low R:FR
a
a
a
a
a
a
a
a
a
a
a
a
a
a
a
a
ab
ab
b
a
a
a
a
a
a
a
a
ab
ab
a
b
a
ab
a
a
### Chart
| Category | | | |
|---|---|---|---|
| Light | 7.114755555555555 | 6.460665555555555 | 5.515537777777777 |
| 2 h Dark | 7.084355555555557 | 6.273835555555556 | 4.989114444444444 |
| 4 h Dark | 7.371391111111112 | 5.992836666666666 | 5.345508749999999 |
| 6 h Dark | 6.528361111111111 | 6.228031111111111 | 5.369023333333334 |a
a
a
a
a
a
a
ab
a
a
a
a
a
a
a
b
a
