# Supplementary material for: Plant competition cues activate a singlet oxygen signaling pathway in Arabidopsis thaliana
Source: Front Plant Sci. 2024 Aug 20;15:964476. doi: 10.3389/fpls.2024.964476 (PMC11368760; doi:10.3389/fpls.2024.964476)
Supplement: Supplementary file 8 [file Presentation4.pptx]

## Slide 1
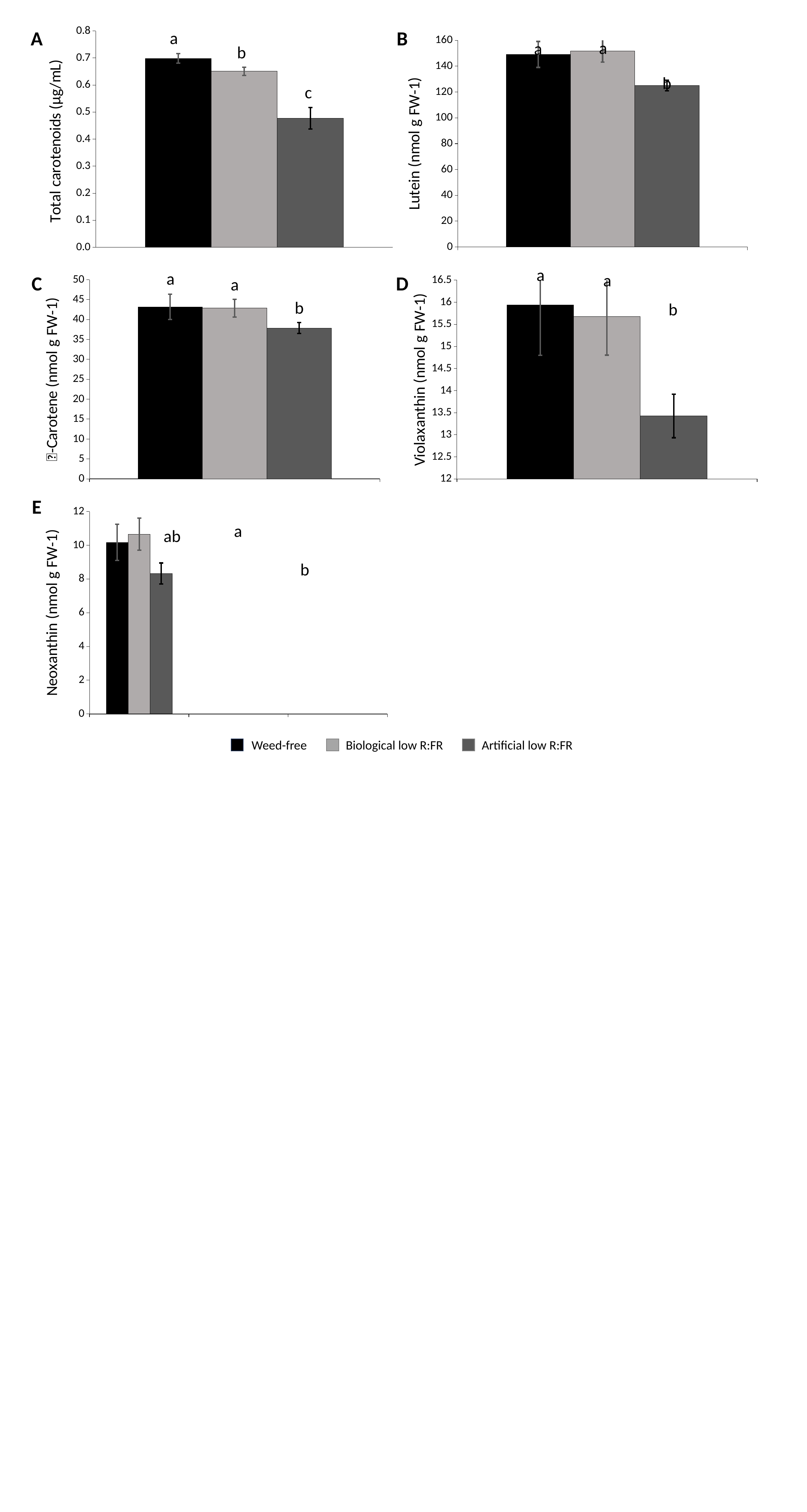

### Chart
| Category | | | |
|---|---|---|---|B
A
### Chart
| Category | | | |
|---|---|---|---|b
c
C
D
### Chart
| Category | | | |
|---|---|---|---|E
[unsupported chart]
Weed-free
Biological low R:FR
Artificial low R:FR
a
a
a
b
a
### Chart
| Category | | | |
|---|---|---|---|a
a
a
b
b
a
ab
b
