# Supplementary material for: Plant competition cues activate a singlet oxygen signaling pathway in Arabidopsis thaliana
Source: Front Plant Sci. 2024 Aug 20;15:964476. doi: 10.3389/fpls.2024.964476 (PMC11368760; doi:10.3389/fpls.2024.964476)
Supplement: Supplementary file 9 [file Presentation5.pptx]

## Slide 1
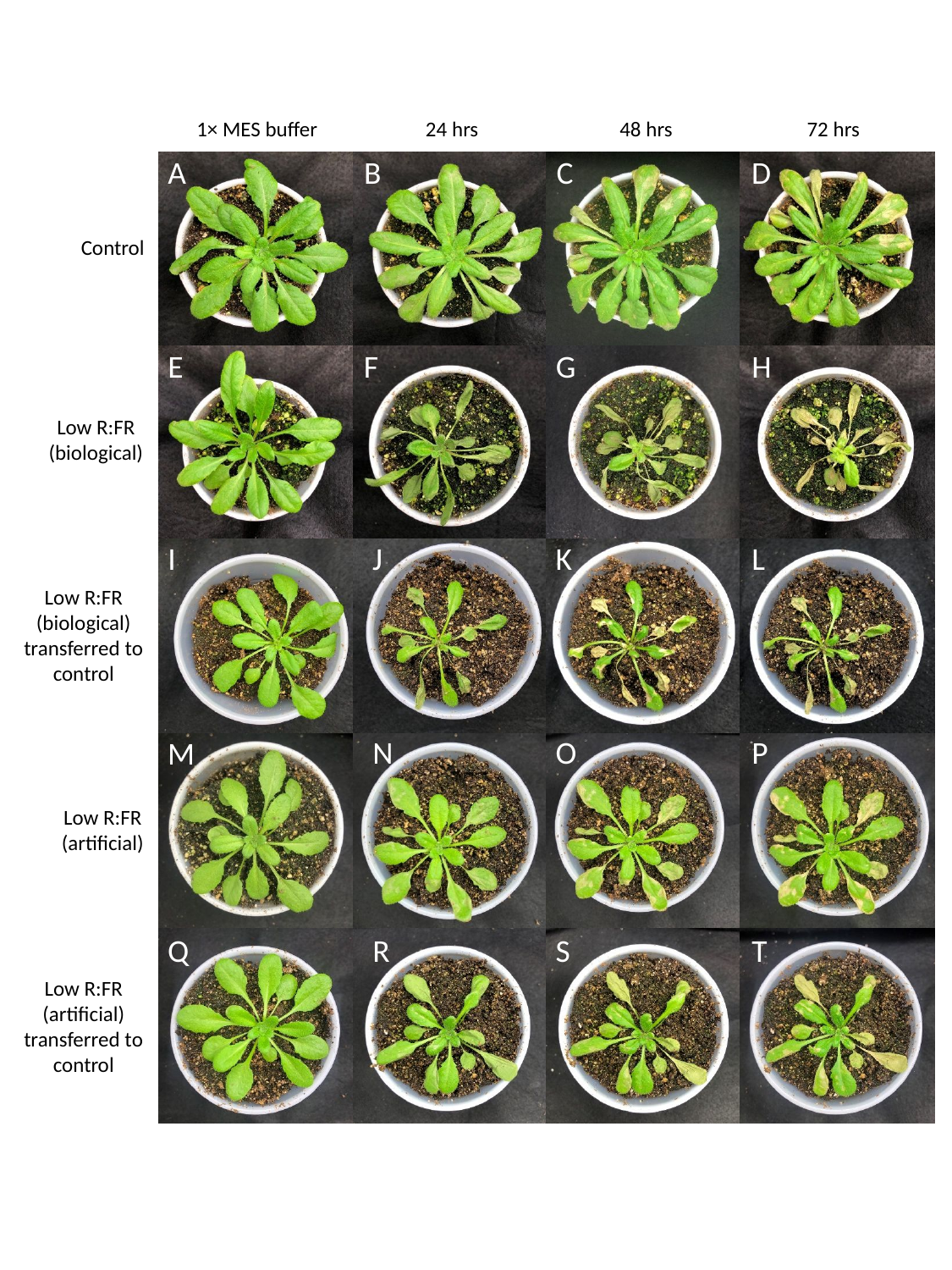

1× MES buffer
24 hrs
48 hrs
72 hrs
A
B
C
D
Control
E
G
H
F
Low R:FR
(biological)
J
L
I
K
Low R:FR
(biological)
transferred to
control
N
O
P
M
Low R:FR
(artificial)
T
Q
R
S
Low R:FR
(artificial)
transferred to
control
