# Supplementary material for: Plant competition cues activate a singlet oxygen signaling pathway in Arabidopsis thaliana
Source: Front Plant Sci. 2024 Aug 20;15:964476. doi: 10.3389/fpls.2024.964476 (PMC11368760; doi:10.3389/fpls.2024.964476)
Supplement: Supplementary file 10 [file Presentation6.pptx]

## Slide 1
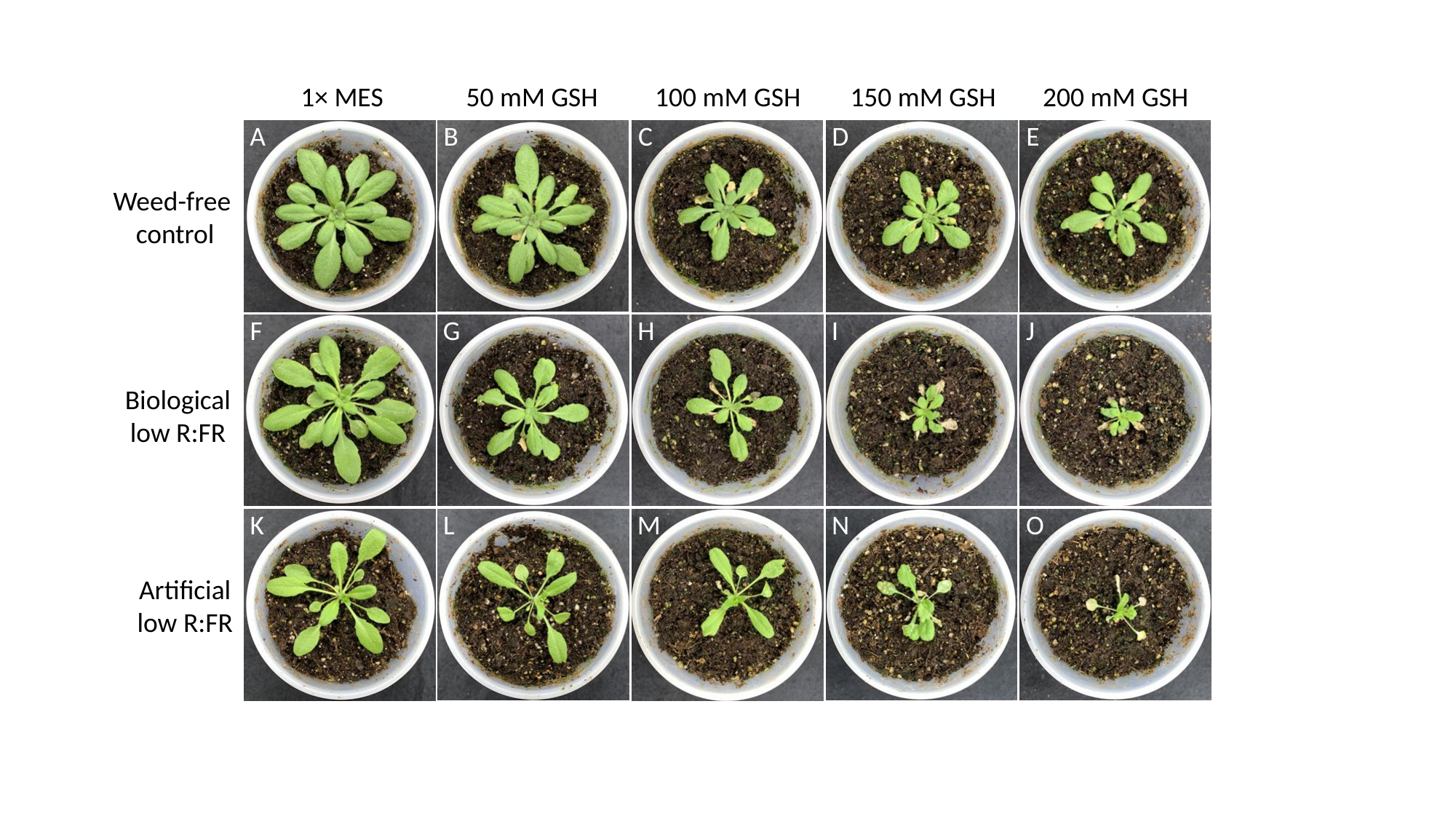

1× MES
50 mM GSH
100 mM GSH
150 mM GSH
200 mM GSH
A
D
B
C
E
Weed-free
control
F
G
H
I
J
Biological
low R:FR
L
K
M
N
O
Artificial
low R:FR
