# Supplementary material for: Plant competition cues activate a singlet oxygen signaling pathway in Arabidopsis thaliana
Source: Front Plant Sci. 2024 Aug 20;15:964476. doi: 10.3389/fpls.2024.964476 (PMC11368760; doi:10.3389/fpls.2024.964476)
Supplement: Supplementary file 11 [file Presentation7.pptx]

## Slide 1
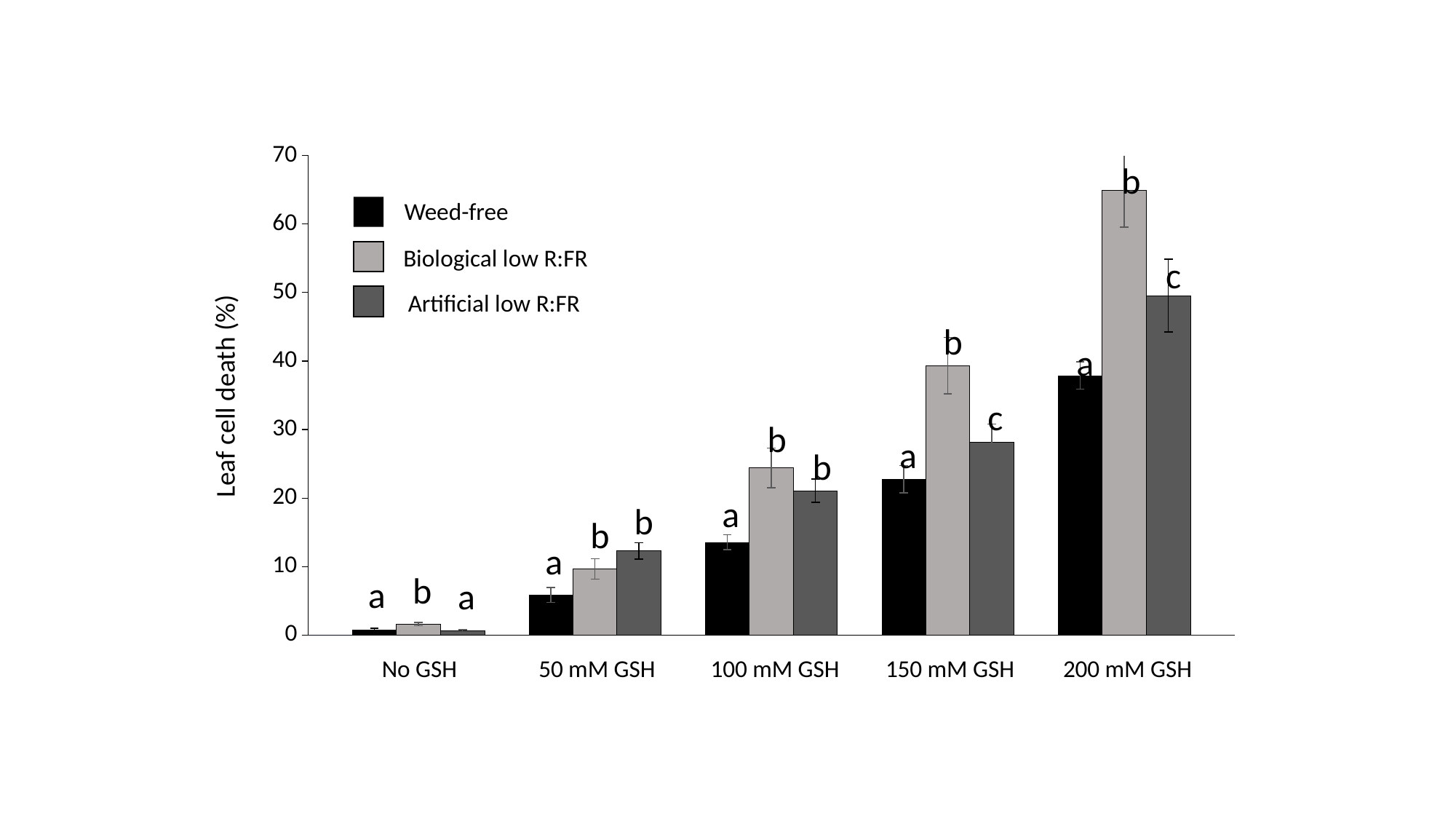

### Chart
| Category | | | | | | | | | | | | | | | | | | | | | |
|---|---|---|---|---|---|---|---|---|---|---|---|---|---|---|---|---|---|---|---|---|---|b
Weed-free
Biological low R:FR
c
Artificial low R:FR
b
Leaf cell death (%)
c
b
b
b
b
b
No GSH
50 mM GSH
100 mM GSH
150 mM GSH
200 mM GSH
a
a
a
a
a
a
