# Supplementary material for: Plant competition cues activate a singlet oxygen signaling pathway in Arabidopsis thaliana
Source: Front Plant Sci. 2024 Aug 20;15:964476. doi: 10.3389/fpls.2024.964476 (PMC11368760; doi:10.3389/fpls.2024.964476)
Supplement: Supplementary file 12 [file Presentation8.pptx]

## Slide 1
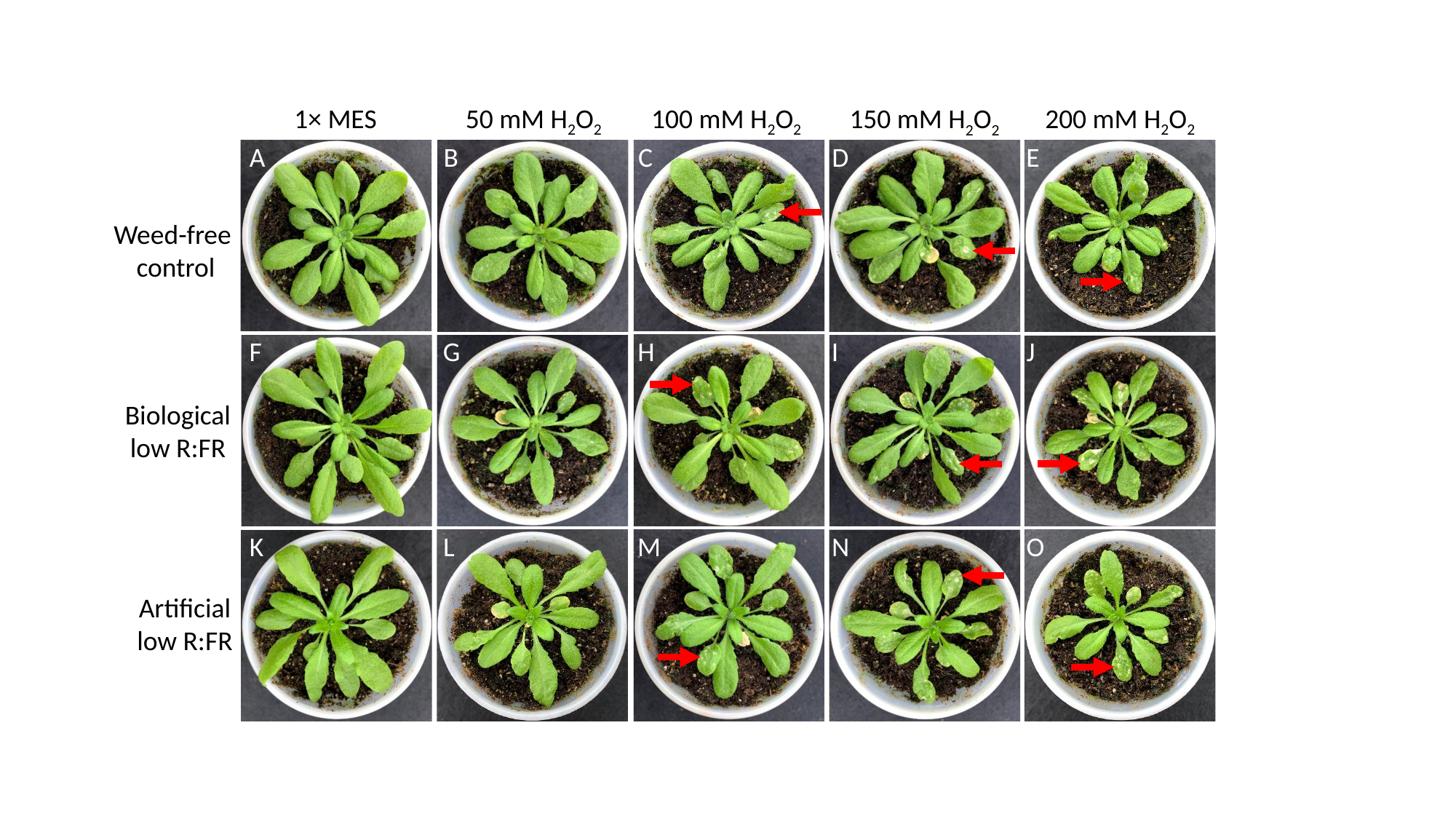

50 mM H2O2
100 mM H2O2
200 mM H2O2
1× MES
150 mM H2O2
D
B
C
E
A
Weed-free
control
G
H
I
J
F
Biological
low R:FR
L
M
N
O
K
Artificial
low R:FR
