# Supplementary figures and images for: Plant competition cues activate a singlet oxygen signaling pathway in Arabidopsis thaliana
Source: Front Plant Sci. 2024 Aug 20;15:964476. doi: 10.3389/fpls.2024.964476 (PMC11368760; doi:10.3389/fpls.2024.964476)

## Slide 1
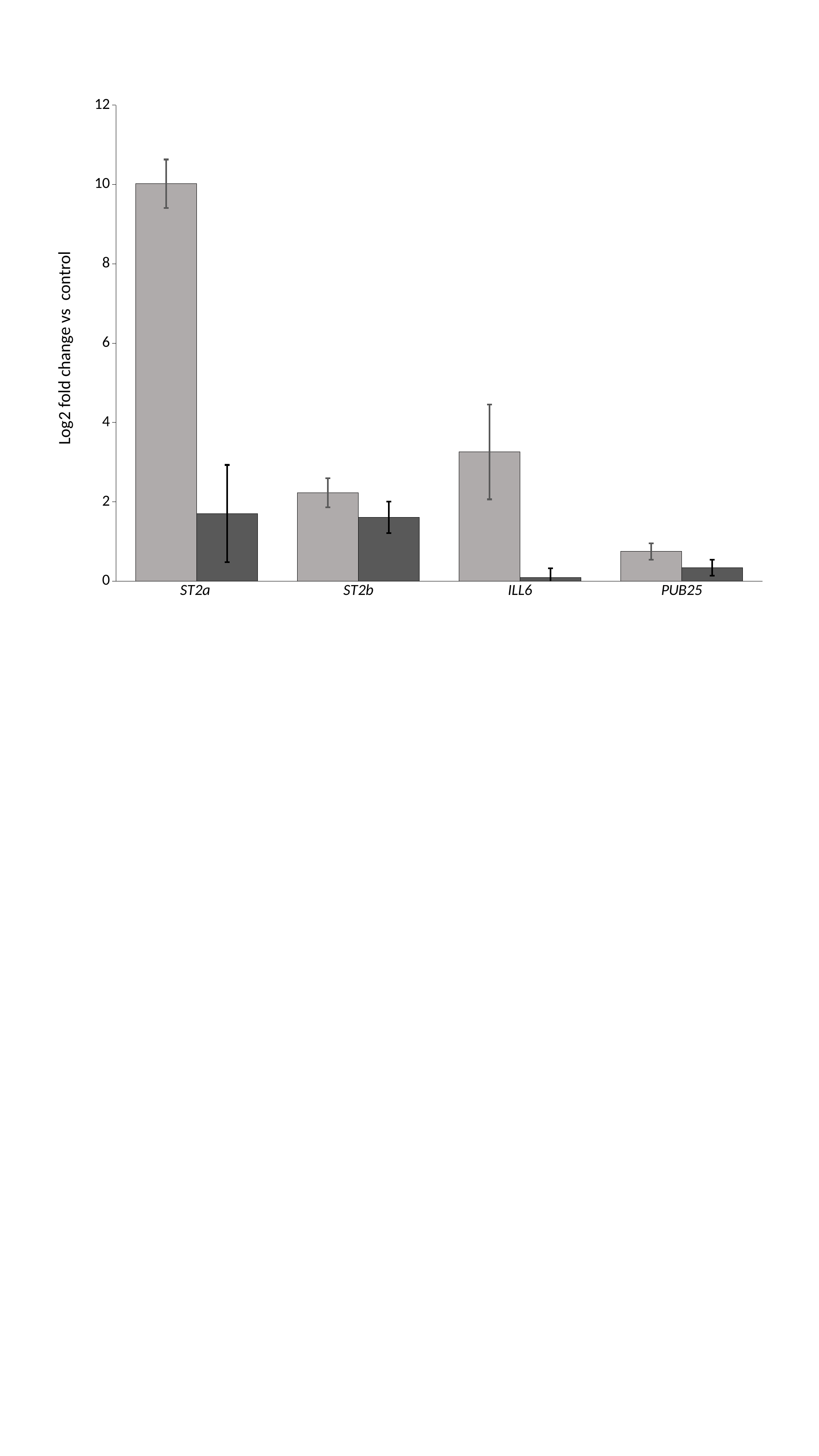

### Chart
| Category | | |
|---|---|---|
| ST2a | 10.018867807816912 | 1.7040147835123027 |
| ST2b | 2.2289618815051213 | 1.6072291340710894 |
| ILL6 | 3.2576142 | 0.091305665 |
| PUB25 | 0.74686285 | 0.34014058 |

Supplement: Supplementary file 13 [file Presentation9.pptx]
